# Differentiating Pulmonary Nodule Malignancy Using Exhaled Volatile Organic Compounds: A Prospective Observational Study

**DOI:** 10.1002/cam4.70545

**Published:** 2025-01-07

**Authors:** Guangyu Lu, Zhixia Su, Xiaoping Yu, Yuhang He, Taining Sha, Kai Yan, Hong Guo, Yujian Tao, Liting Liao, Yanyan Zhang, Guotao Lu, Weijuan Gong

**Affiliations:** ^1^ Department of Health Management Center Affiliated Hospital of Yangzhou University, Yangzhou University Yangzhou Jiangsu China; ^2^ School of Public Health Medical College of Yangzhou University, Yangzhou University Yangzhou Jiangsu China; ^3^ School of Nursing Medical College of Yangzhou University, Yangzhou University Yangzhou Jiangsu China; ^4^ Department of Thoracic Surgery Affiliated Hospital of Yangzhou University, Yangzhou University Yangzhou Jiangsu China; ^5^ Department of Respiratory and Critical Care Medicine Affiliated Hospital of Yangzhou University, Yangzhou University Yangzhou Jiangsu China; ^6^ Department of Basic Medicine Medical College of Yangzhou University, Yangzhou University Yangzhou Jiangsu China; ^7^ Testing Center of Yangzhou University, Yangzhou University Yangzhou Jiangsu China; ^8^ Yangzhou Key Laboratory of Pancreatic Disease Institute of Digestive Diseases, Affiliated Hospital of Yangzhou University, Yangzhou University Yangzhou Jiangsu China; ^9^ Pancreatic Center, Department of Gastroenterology Affiliated Hospital of Yangzhou University, Yangzhou University Yangzhou Jiangsu China

**Keywords:** breath biomarkers, malignancy risk, pulmonary nodules, volatile organic compounds

## Abstract

**Background:**

Advances in imaging technology have enhanced the detection of pulmonary nodules. However, determining malignancy often requires invasive procedures or repeated radiation exposure, underscoring the need for safer, noninvasive diagnostic alternatives. Analyzing exhaled volatile organic compounds (VOCs) shows promise, yet its effectiveness in assessing the malignancy of pulmonary nodules remains underexplored.

**Methods:**

Employing a prospective study design from June 2023 to January 2024 at the Affiliated Hospital of Yangzhou University, we assessed the malignancy of pulmonary nodules using the Mayo Clinic model and collected exhaled breath samples alongside lifestyle and health examination data. We applied five machine learning (ML) algorithms to develop predictive models which were evaluated using area under the curve (AUC), sensitivity, specificity, and other relevant metrics.

**Results:**

A total of 267 participants were enrolled, including 210 with low‐risk and 57 with moderate‐risk pulmonary nodules. Univariate analysis identified 11 exhaled VOCs associated with nodule malignancy, alongside two lifestyle factors (smoke index and sites of tobacco smoke inhalation) and one clinical metric (nodule diameter) as independent predictors for moderate‐risk nodules. The logistic regression model integrating lifestyle and health data achieved an AUC of 0.91 (95% CI: 0.8611–0.9658), while the random forest model incorporating exhaled VOCs achieved an AUC of 0.99 (95% CI: 0.974–1.00). Calibration curves indicated strong concordance between predicted and observed risks. Decision curve analysis confirmed the net benefit of these models over traditional methods. A nomogram was developed to aid clinicians in assessing nodule malignancy based on VOCs, lifestyle, and health data.

**Conclusions:**

The integration of ML algorithms with exhaled biomarkers and clinical data provides a robust framework for noninvasive assessment of pulmonary nodules. These models offer a safer alternative to traditional methods and may enhance early detection and management of pulmonary nodules. Further validation through larger, multicenter studies is necessary to establish their generalizability.

**Trial Registration:** Number ChiCTR2400081283

## 1 Introduction

Pulmonary nodules are defined as spherical lesions within the lung parenchyma, clearly delineated with a diameter of 3 cm or less [1]. With the increased accessibility and utilization of computed tomography (CT) scans, the incidental detection of pulmonary nodules has become significantly more common [2, 3, 4]. For example, approximately 30% of diagnostic chest CT in the United States demonstrate an incidental pulmonary nodule every year [5]. Following the expansion of the United States Preventive Services Task Force criteria in 2021, the estimated number of individuals eligible for lung cancer screening has doubled from 8 to 15 million, therefore, the number of pulmonary nodules detected through screening is expected to rise considerably [6, 7]. While most pulmonary nodules are benign, the potential for malignancy necessitates rigorous diagnostic protocols. Conventional methods for assessing malignancy for pulmonary nodules include CT, X‐ray, sputum cytology, and biopsy [8, 9, 10, 11, 12].

Chest radiography and sputum cytology have been used for assessing malignancy for pulmonary nodules since the 1970s. However, the former can expose patients to additional radiation, and the sensitivity levels of these modalities are low [13, 14, 15]. Although invasive surgical methods, including biopsy and surgical interventions, can be performed for improving diagnostic accuracy, it can lead to unnecessary costs and morbidity, as it may result in the surgical resection of tumors that exhibit no clinical symptoms [15]. Therefore, there is a compelling need for a rapid, noninvasive, and effective diagnostic alternative [8].

Breath analysis, leveraging exhaled volatile organic compounds (VOCs), presents a promising solution for diagnosing lung disorders [16, 17, 18]. Breath is a complex mixture of gases and aerosols that contains hundreds of VOCs, which serve as helpful indicators of various lung conditions [19, 20]. The analysis of exhaled VOCs offers several advantages, including noninvasiveness, ease of performance, and the ability to detect both early and advanced stages of diseases [21, 22]. Extensive research is currently underway to explore exhaled VOCs‐based diagnosis for various ailments, including lung cancer, ovarian cancer, COPD, tuberculosis, pneumonia, asthma, cystic fibrosis, etc. [23, 24, 25, 26, 27, 28].

Owning to pulmonary nodules might be the initial radiologic manifestation of lung cancers [29, 30], the mechanism of these nodules could involve the metabolic changes associated with cancer. Previous study has demonstrated that the altered genome and transcriptome during carcinogenesis and progression will lead to dysregulated metabolic pathways and the accumulation of aberrant metabolites [31]. Among numerous metabolites, lung cancer‐derived VOCs can diffuse into alveoli and can be detected in exhaled breath [16]. These VOCs can reflect the metabolic state of individuals and can be used as biomarkers for lung cancer [17, 32]. Therefore, this method could potentially transform how we assess the malignancy of pulmonary nodules.

Our study explores the potential of exhaled VOCs linked to the malignancy of pulmonary nodules. By analyzing breath samples from individuals with pulmonary nodules stratified by their risk levels, we employed five distinct machine learning (ML) algorithms to create models that integrate epidemiological data, health examination outcomes, and VOC biomarkers. These models aim to predict the likelihood of malignancy in pulmonary nodules. We also developed a prediction nomogram to improve the clinical assessment of pulmonary nodules in asymptomatic individuals. This may improve the assessment process, offering a noninvasive, accurate, and efficient method for evaluating pulmonary nodules.

## 2 Methods

### 2.1 Study Design and Population

The prospective study was registered in the Chinese Clinical Trial Registry (Clinical Trials Registration Number ChiCTR2400081283). This study was conducted at the Health Management Center of the Affiliated Hospital of Yangzhou University, China from June 1, 2023 to January 31, 2024. Participants were eligible for inclusion if they were aged above 45 years and scheduled to undergo routine low‐dose computed tomography (LDCT) scans. Individuals were consecutively recruited to ensure a representative sample of the population attending the center.

The exclusion criteria were as follows: (1) participants were unable to understand or cooperate with the breath collection process; (2) participants had cancer histories; (3) participants had a history of airway inflammatory or lung infection in the past 3 months; (4) participants had liver or kidney dysfunction, asthma, COPD, diabetes, and tuberculosis that may change the exhaled breath profile; (5) participants were lack of planned breath sample; (6) participants were unwilling to provide written informed consent to participate; and (7) pregnant and lactating women, as the safety of LDCT in these populations cannot be guaranteed.

### 2.2 Definition of Outcome

The evaluation of the malignancy of pulmonary nodules in study participants was systematically conducted using the Mayo Clinic model, as detailed in the Supplemental Methods (Section A) of our documentation. This model incorporates a range of clinical, radiographic, and demographic factors to estimate the probability of malignancy in pulmonary nodules detected on CT scans.

The initial risk assessment was performed independently by two researchers (Zhixia Su and Taining Sha), each trained in the Mayo Clinic model's application. This dual‐assessment approach was employed to minimize subjective bias and enhance the robustness of the risk categorization. Following the independent assessments, the results were reviewed by an experienced thoracic expert (Dr. Yujian Tao). This review served to resolve any discrepancies between the initial assessments and to finalize the malignancy categorization based on a consensus or the expert's final judgment.

Based on the Mayo Clinic model's output, the probability of nodule malignancy was categorized into three distinct risk levels: [33, 34]

- Low Risk: A malignancy probability of less than 5%. Nodules in this category are typically monitored with periodic imaging to detect any changes in size or appearance.
- Moderate Risk: A malignancy probability between 5% and 65%. Nodules falling within this range often require further diagnostic investigation, such as positron emission computed tomography (PET) scans or biopsy, depending on individual patient factors and nodule characteristics.
- High Risk: A malignancy probability greater than 65%. These nodules are prioritized for immediate diagnostic intervention to ascertain malignancy through invasive procedures or advanced imaging techniques.

### 2.3 Sample Size Calculation

In this study, the sample size calculation was guided by requirements for logistic regression (LR) models, which are commonly used in developing prediction models for binary or time‐to‐event outcomes. The rule of thumb for LR is to have at least 10 events per predictor parameter to ensure robust statistical power and minimize the risk of overfitting. Given the use of 14 independent variables in the model, and based on the established rule, the minimum number of events needed was calculated as 140 (14 variables × 10 events per variable). Assuming a prevalence rate of 55.9% for the detection of pulmonary nodules in chest CT scans, the total sample size required was estimated using the formula:

$$N=\frac{14\times 10}{0.559}\approx 250$$

To accommodate this calculation and potential nonresponse or data loss, 267 participants were enrolled. This figure exceeds the calculated minimum, thereby satisfying the statistical requirements for all analytical algorithms employed in the study.

### 2.4 Thoracic CT Scans

Participants underwent routine CT scans as part of the study protocol. Prior to imaging, all participants received instruction on breathing techniques and were scanned in the supine position using a deep inspiration breath‐hold approach. Scans encompassed the region from the top of the skull to the base. The CT parameters were set at 120 kV for voltage, 250 mA for tube current, with a slice thickness of 5 mm, and an image resolution of 512 × 512 pixels. Radiological assessments were conducted by two experienced chest radiologists with over 10 years of experience, via consensus evaluation.

### 2.5 Data Collection

A questionnaire was developed to gather epidemiological data, following a structured panel discussion involving experts in clinical epidemiologist and doctors at the Health Management Center. This questionnaire captured key demographic characteristics (e.g., age and gender) and lifestyle factors (Supplemental Methods, Section B). Health‐related data, radiological signs, and laboratory results were meticulously extracted from the health management records. Data collection was independently conducted by researchers and cross‐check to ensure reliability. Any discrepancies encountered were collaboratively discussed and resolved until a consensus was reached among the researchers. Discrepancies were resolved through joint discussion by the researchers (Weijuan Gong Yujian Tao, and Xiaoping Yu) until consensus was reached.

### 2.6 Breath Sample Collection

Breath samples were collected from participants in a controlled environment using Tedlar bags (Inner Mongolia Ailite Environmental Protection Technology Co. China), immediately following a standard protocol to minimize sample variability. Prior to sampling, participants rinsed their mouths using the same brand of mouthwash (Saky, China) and then performed a deep inhalation through the nose and a complete exhalation into the bags. A total volume of 1000 mL of breath was collected per participant. To preserve the integrity of the VOCs, exhaled breath was promptly transferred to a sorbent tube containing Tenax GR and Carbopack B (Markes International Ltd., UK) using a pump set at a flow rate of 250 mL/min. Collection occurred on the morning of a scheduled health examination, with all participants fasting for at least 8 h and avoiding spicy foods, alcohol, and coffee the previous evening.

### 2.7 TD‐GC × GC‐TOF MS Analysis and Feature Identification

Breath analysis and feature identification were conducted using a comprehensive two‐dimensional gas chromatography × gas chromatography‐time‐of‐flight mass spectrometry (TD‐GC × GC‐TOF MS) system. Detailed instrumentation settings and parameters are provided in the Supplemental Methods, Section C. Data acquisition and processing were performed using ChromSpace software version 2.1 (SepSolve Analytical Ltd., UK). This software facilitated peak detection, mass deconvolution, peak integration, and library searching against the National Institute of Standards and Technology (NIST 2014) mass spectral libraries, with a minimum acceptable match factor of 700. The statistical comparison tool within ChromSpace 2.1 was utilized to align two‐dimensional chromatograms and to construct comprehensive peak tables, including all detected peaks with a signal‐to‐noise ratio exceeding 100. These peak tables were exported as .csv files for subsequent data analysis.

### 2.8 Development and Assessment of the Predictive Models

To select variables for inclusion in predictive models, a systematic literature review was initially conducted to identify candidate predictors. Preliminary univariate analysis assessed the differences in various indicators across groups. To mitigate the influence of features with disproportionately large values, min‐max scaling was applied to normalize all candidate variables to a range between 0 and 1.

Binary logistic regression (LR) analysis was then employed to estimate the odds ratios (ORs) and their 95% confidence intervals (CIs) for these variables. Furthermore, the least absolute shrinkage and selection operator (LASSO) regression, utilizing L1 regularization, was used to determine the inclusion and exclusion of variables based on the magnitude of their coefficients. Variables with zero coefficients were excluded, while those with nonzero coefficients were retained for model development.

Three model configurations were developed based on different combinations of variables:

Model 1: A lifestyle‐based model incorporating demographic characteristics and lifestyle factors.

Model 2: A health examination‐lifestyle‐based model, which adds health examination data to the variables used in the lifestyle‐based model.

Model 3: A breathomics‐health examination‐lifestyle‐based model, which includes exhaled VOCs in addition to the variables used in the second model.

Machine learning algorithms, including logistic regression (LR), decision tree (DT), random forest (RF), K‐nearest neighbors (KNN), and support vector machine (SVM), were utilized as classifiers to predict the probability of malignancy in participants with pulmonary nodules, which gives promising results in the analysis of VOC analysis of exhaled breath [35, 36, 37, 38]. The performance of these models was evaluated using sensitivity, specificity, accuracy, positive predictive value (PPV), negative predictive value (NPV), and the area under the receiver operating characteristic curve (AUC). DeLong's test was employed to compare the AUCs of different models, and calibration curve analysis along with decision curve analysis (DCA) was conducted to assess the predictive performance of the models.

### 2.9 Statistical Analysis

Participants' data were classified into continuous or categorical variables. The normality of continuous variables was tested using the Kolmogorov–Smirnov test. Normally distributed continuous variables were expressed as mean ± standard deviation and compared using the *t*‐test. For continuous variables that did not meet normality assumptions, the Mann–Whitney *U* test was applied, and results were presented as medians with interquartile ranges. Categorical variables were expressed as counts (percentages) and analyzed using the Chi‐square test, Fisher's exact test, or Poisson regression analysis, tailored to fit the data structure and sample size. Statistical significance was established at a two‐sided P‐value of less than 0.05 unless otherwise specified. Statistical analyses were performed using R software version 4.3.2 and MedCalc software. The glmnet package was utilized for building the LASSO model. AUCs were drawn using the pROC package, while the ggplot2 package facilitated the creation of calibration curves. DCA was conducted using the dcurves and rmda packages. Handling of missing data was stratified based on their proportions: variables with less than 5% missing data were interpolated using mean or median values; those with 5% to 20% missing data underwent multiple imputation; and variables with more than 20% missing data were excluded from the analysis.

### 2.10 Ethics Statement

The institutional review board of the Affiliated Hospital of Yangzhou University approved this study (2022‐YKL06‐SKJ005). All participants were informed of the study protocol, and written consent was obtained before participating in the study.

## 3 Results

### 3.1 Study Participants and Characteristics

**FIGURE 1:**
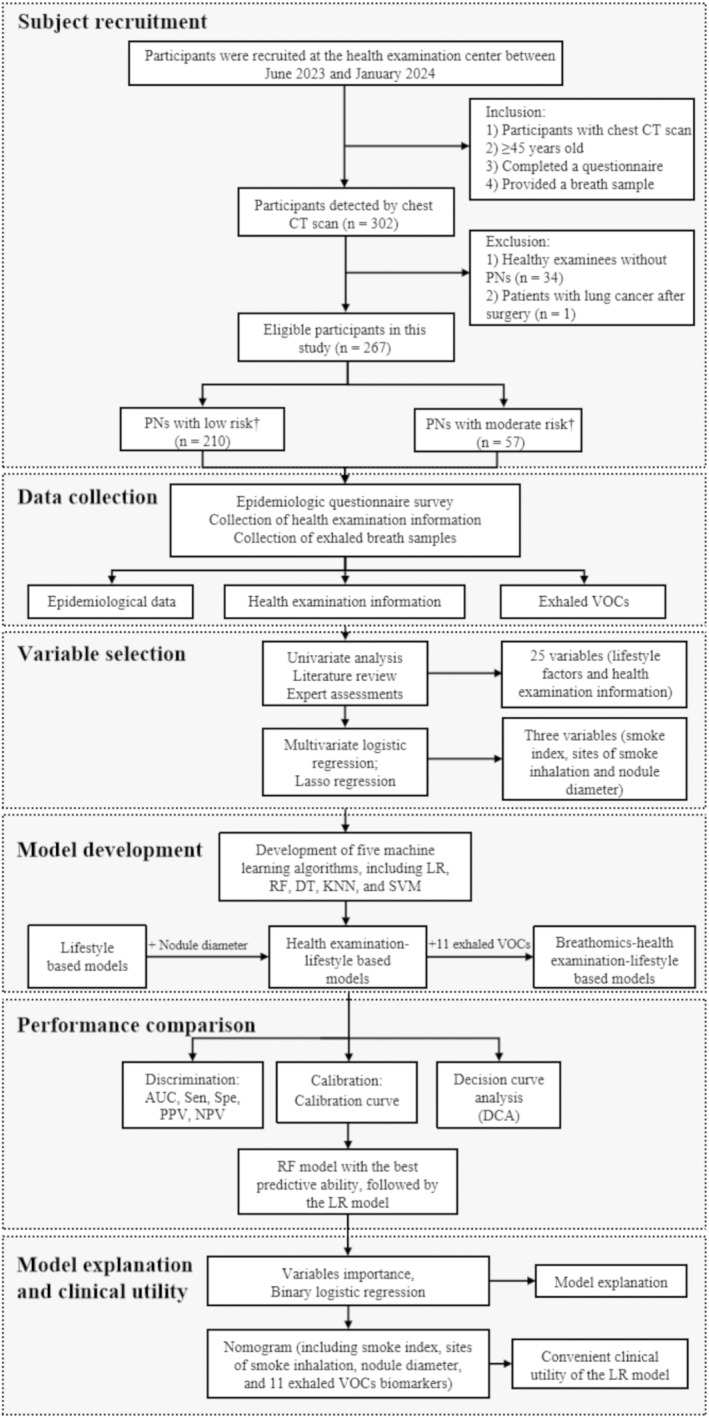
Flowchart of study design. † The assessment of the probability of malignancy in individuals with pulmonary nodules was performed according to the Mayo Clinic model. Epidemiological data, including demographic characteristics and lifestyle factors, were collected by using an adaptive questionnaire. Health examination data, including radiological signs and laboratory findings, were extracted from the health examination management records. AUC: area under the curve; CT: computed tomography; DT: decision tree model; KNN: K‐nearest neighbor model; LR: logistic regression model; NPV: negative predictive value; PNs: pulmonary nodules; PPV: positive predictive value; RF: random forest model; Sen: sensitivity; Spe: specificity; SVM: support vector machine model; VOCs: volatile organic compounds.

A total of 267 participants were enrolled in this study (Figure 1), comprising 169 males (63%) and 98 females (37%). The majority, 210 participants (80%), were categorized into the low‐risk group, while 57 participants (21.3%) fell into the moderate‐risk group. Analytical processing of exhaled VOCs yielded 1166 entities, with 139 receiving confirmed annotations from the Human Metabolome Database (HMDB). Detailed comparisons of demographic characteristics, lifestyle factors, health examination results, and exhaled VOC indicators between the groups are presented in Tables 1 and 2, and Tables S1 and S2.

**TABLE 1:** Demographics, lifestyle factors, and health examination data of participants with pulmonary nodules in low‐ and moderate‐risk groups (*p* ≤ 0.05).^a^

Variables	Total (*n* = 267)	Low‐risk group (*n* = 210)	Moderate‐risk group (*n* = 57)	*p* value
Gender, *n* (%)				
Male	169 (63)	119 (57)	50 (88)	**< 0.001**
Female	98 (37)	91 (43)	7 (12)	
Sites of tobacco smoke inhalation, *n* (%)				
Never smoke	218 (82)	199 (95)	19 (33)	**< 0.001**
Inhaled into mouth	21 (8)	5 (2)	16 (28)	
Inhaled into throat	2 (1)	0 (0)	2 (4)	
Inhaled into lung	26 (10)	6 (3)	20 (35)	
Smoke index, *n* (%)				
Mild:0–10 pack‐years	222 (83)	202 (96)	20 (35)	**< 0.001**
Moderate:10–20 pack‐years	25 (9)	2 (1)	23 (40)	
Severe:> 20 pack‐years	20 (7)	6 (3)	14 (25)	
Exposure to secondhand smoke in the workplace, *n* (%)				
Yes	78 (29)	53 (25)	25 (44)	**0.01**
No	189 (71)	157 (75)	32 (56)	
Alcohol intake frequency, *n* (%)				
Never	195 (73)	166 (79)	29 (51)	**< 0.001**
≤ 1 time/week	30 (11)	20 (10)	10 (18)	
2–6 times/week	37 (14)	21 (10)	16 (28)	
Everyday	3 (1)	1 (0)	2 (4)	
Abstinent from alcohol	2 (1)	2 (1)	0 (0)	
Tea consumption, *n* (%)				
Yes	139 (52)	100 (48)	39 (68)	**0.008**
No	128 (48)	110 (52)	18 (32)	
Nodule diameter (cm)^c^	0.5 (0.4, 0.6)	0.5 (0.4, 0.5)	0.5 (0.4, 0.7)	**0.002**
GGT^c^	26.1 (16.9, 39.1)	23.2 (15.7, 37.48)	31 (23.9, 46.2)	**< 0.001**
AST^c^	20.8 (17.85, 24.55)	20.3 (17.62, 24.23)	22.5 (19.3, 25.4)	**0.012**
Monocyte^c^	0.38 (0.31, 0.48)	0.37 (0.3, 0.46)	0.44 (0.37, 0.54)	**< 0.001**
HCT^b^	44.07 ± 3.88	43.67 ± 3.93	45.57 ± 3.34	**< 0.001**
RBC^b^	4.8 ± 0.42	4.76 ± 0.43	4.92 ± 0.37	**0.005**
Hemoglobin^c^	147 (136, 155)	145.98 (135, 154)	151 (145, 159)	**< 0.001**
WBC^c^	6.14 (5.22, 7.07)	6.04 (5.14, 6.96)	6.4 (5.9, 7.29)	**0.016**
NE^c^	3.24 (2.66, 3.87)	3.14 (2.54, 3.81)	3.41 (3.11, 4.23)	**0.014**
CEA^c^	1.85 (1.31, 2.41)	1.71 (1.19, 2.28)	2.15 (1.85, 2.57)	**< 0.001**
HDL‐C^c^	1.31 (1.12, 1.52)	1.35 (1.14, 1.56)	1.25 (1.07, 1.38)	**0.003**
TG^c^	1.47 (0.99, 2.14)	1.39 (0.87, 2.08)	1.7 (1.28, 2.33)	**0.016**
AU^c^	321.5 (270.55, 382.8)	314.55 (263.65, 378.4)	331.9 (302.3, 389.8)	**0.021**
CR^b^	75.52 ± 16.62	73.74 ± 16.94	82.11 ± 13.59	**< 0.001**

*Note:* Bold values represented that *P*‐values were less than 0.05 from the univariate analysis, aiming to highlight that these variables have statistical significance between two groups.

Abbreviations: AST, aspartate transaminase; AU, aric acid; CEA, carcinoembryonic antigen; CR, creatinine; GGT, gamma glutamyl transferase; HCT, hematocrit; HDL‐C, high‐density lipoprotein cholesterol; NE, neutrophilicgranulocyte; RBC, red blood cells; TG, triglyceride; WBC, white blood cell.

^a^ The assessment of probability of malignancy in individuals with pulmonary nodules was reached according to the Mayo Clinic model.

^b^ Mean ± standard deviation.

^c^ Median (P25, P75).

**TABLE 2:** Exhaled VOCs of participants with pulmonary nodules in low‐ and moderate‐risk groups (*p* ≤ 0.1).^a^

Exhaled VOCs	Total (*n* = 267)	Low‐risk group (*n* = 210)	Moderate‐risk group (*n* = 57)	*p* value
1‐Decanol^b^	0 (0, 0)	0 (0, 0)	0 (0, 264363.2)	0.052
2‐Butenal^d^	0 (0, 9138254.26)	0 (0, 4432026.291)	0 (0, 9138254.26)	**0.026**
2‐Naphthalenol^c^	0 (0, 1286188.8838)	0 (0, 1473604.82655)	0 (0, 955929.84923)	**0.016**
Acetaldehyde^b^	0 (0, 865428.48)	0 (0, 1658178.63)	0 (0, 0)	0.085
Benzoic acid^b^	5796904.58 (3874596.38, 8821848.94)	5509199.03 (3797335.86, 8589702.26)	6859890.65 (4361591.63, 10315932.88)	0.088
Dimethyl sulfide^c^	0 (0, 3681947.221)	0 (0, 0)	0 (0, 3681947.221)	0.073
Furan^b^	0 (0, 2465299.37)	0 (0, 1901160.45)	1658920.99 (0, 3490469.22)	**0.003**
l‐Menthone^c^	0 (0, 735144.93516)	0 (0, 1292211.87505)	0 (0, 70758.77928)	0.088
Methyl‐2‐thiophene^b^ carboxylate^c^	0 (0, 0)	0 (0, 0)	0 (0, 428010.17748)	0.073
Naphthalene^b^	0 (0, 1062199.39)	0 (0, 533109.2)	0 (0, 1574990.18)	0.066
Octanal^b^	0 (0, 0)	0 (0, 0)	0 (0, 263589.18)	**0.044**

*Note:* Bold values represented that *P*‐values were less than 0.05 from the univariate analysis, aiming to highlight that these variables have statistical significance between two groups.

Abbreviation: VOCs, Volatile organic compounds.

^a^ The assessment of probability of malignancy in individuals with pulmonary nodules was reached according to the Mayo Clinic model. Skewed distributed quantitative data were presented as median (percentile range) and compared between groups using the Mann–Whitney *U* test.

^b^ Median (P25, P75).

^c^ Median (P5, P95).

^d^ Median (P1, P100).

Biochemical analysis revealed significant differences between the moderate‐risk and low‐risk groups in several markers (*p* < 0.05). Specifically, concentrations of gamma‐glutamyl transferase (GGT), aspartate aminotransferase (AST), monocytes, hematocrit (HCT), red blood cells (RBC), hemoglobin, white blood cells (WBC), neutrophil (NE), carcinoembryonic antigen (CEA), triglycerides (TG), uric acid (UA), and C‐reactive protein (CR) were all notably higher in the moderate‐risk group (*p* < 0.05). In contrast, the concentration of high‐density lipoprotein cholesterol (HDL‐C) was significantly lower in this group (Table 1).

In terms of VOCs exhaled by participants, concentrations of 2‐butenal, octanal, naphthalene, benzoic acid, 1‐decanol, dimethyl sulfide, furan, and methyl‐2‐thiophene carboxylate were significantly higher in the moderate‐risk group (*p* < 0.1). Conversely, levels of acetaldehyde, 2‐naphthalenol, and I‐menthone were lower in this group (Table 2).

### 3.2 Development of the Predictive Models

In our analysis, 25 lifestyle and health examination variables were considered, of which smoke index, sites of tobacco smoke inhalation, and nodule diameter emerged as independent predictors for moderate‐risk pulmonary nodules as identified through binary logistic regression and LASSO regression analyses (Figure S1, Tables S3 and S5). To enhance model performance, exhaled VOC variables were incorporated, and 11 VOCs were identified as critical for predicting the malignancy of pulmonary nodules, which include 2‐butenal, acetaldehyde, octanal, 2‐naphthalenol, naphthalene, benzoic acid, 1‐decanol, dimethyl sulfide, furan, methyl‐2‐thiophene carboxylate, and l‐menthone. Five different ML models, including LR, DT, RF, KNN, and SVM, were developed based on varying combinations of these indicators (Tables S4 and S6).

### 3.3 Performance of the Predictive Models

The lifestyle‐based ML models showed varied performance levels. The LR model significantly outperformed others, exhibiting an AUC of 0.85 (95% CI: 0.7882–0.9083), with sensitivity at 96.19%, specificity at 59.65%, and overall accuracy of 0.88 (Table 3, Figure S3a). With the addition of health examination variables to the lifestyle factors, the LR model's performance further improved, achieving an AUC of 0.91 (95% CI: 0.8611–0.9658), sensitivity of 95.24%, specificity of 70.18%, and accuracy of 0.90 (Table 3, Figure S3b). Calibration curves for both models approached the ideal diagonal (Figures S4a and S4b), indicating strong predictive alignment.

**TABLE 3:** Performance comparison of selected ML algorithms for three predictive models.

Models	ML algorithms	AUC (95%CI)	Sen	Spe	Acc	PPV	NPV
Lifestyle‐based models	LR^a^	0.8482 (0.7882–0.9083)	0.9619	0.5965	0.8839	0.8978	0.8095
	DT	0.8452 (0.7853–0.9051)	0.9476	0.7368	0.9026	0.9299	0.7925
	RF	0.8422 (0.7826–0.9018)	0.9476	0.7368	0.9026	0.9299	0.7925
	KNN	0.8271 (0.7654–0.8887)	0.9524	0.7018	0.8989	0.9217	0.8
	SVM	0.8055 (0.7417–0.8693)	0.9619	0.6491	0.8951	0.9099	0.8222
Health examination‐lifestyle‐based models	LR^a^	0.9135 (0.8611–0.9658)	0.9524	0.7018	0.8989	0.9217	0.8
	DT	0.9021 (0.8513–0.9528)	0.9571	0.7895	0.9213	0.9437	0.8333
	KNN	0.8781 (0.8233–0.9328)	0.9667	0.7895	0.9288	0.9442	0.8654
	RF	0.8677 (0.8105–0.9248)	0.981	0.7544	0.9326	0.9364	0.9149
	SVM	0.7967 (0.7323–0.8612)	0.9619	0.6316	0.8914	0.9058	0.8182
Breathomics‐health examination‐lifestyle‐based models	RF^a^	0.9912 (0.974–1.00)	0.99	0.9825	0.9963	0.9953	0.99
	LR	0.9464 (0.9114–0.9815)	0.9571	0.7368	0.9101	0.9306	0.8235
	DT	0.9021 (0.8513–0.9528)	0.9571	0.7895	0.9213	0.9437	0.8333
	KNN	0.8964 (0.8441–0.9487)	0.9857	0.807	0.9476	0.9495	0.9388
	SVM	0.7115 (0.6434–0.7797)	0.9143	0.5088	0.8277	0.8727	0.617

Abbreviations: Acc, accuracy; AUC, area under the receiver operating characteristic curve; CI, confidence interval; DT, decision tree model; KNN, K‐nearest neighbors model; LR, logistic regression model; ML, machine learning; NPV, negative predictive value; PPV, positive predictive value; RF, random forest model; Sen, sensitivity; Spe, specificity; SVM, support vector machine model.

^a^ Algorithms which achieved the highest performances.

Upon incorporating exhaled VOC data, there was a noticeable enhancement in model efficacy. The RF model notably excelled, reaching an AUC of 0.99 (95%CI: 0.974–1.00), showcasing a substantial increase in model accuracy (Table 3, Figure S3c). Hierarchical analysis of risk factors revealed that smoke index, site of tobacco smoke inhalation, nodule diameter, benzoic acid, and furan were pivotal predictors, crucially impacting the model outcomes (Figure S2). The DCA curve confirmed the high clinical utility of these models, indicating effective risk stratification across a broad probability threshold range (Figure S5). The performance of the ML algorithms for predictive models is summarized in Table 3.

### 3.4 Development of the Nomogram

**FIGURE 2:**
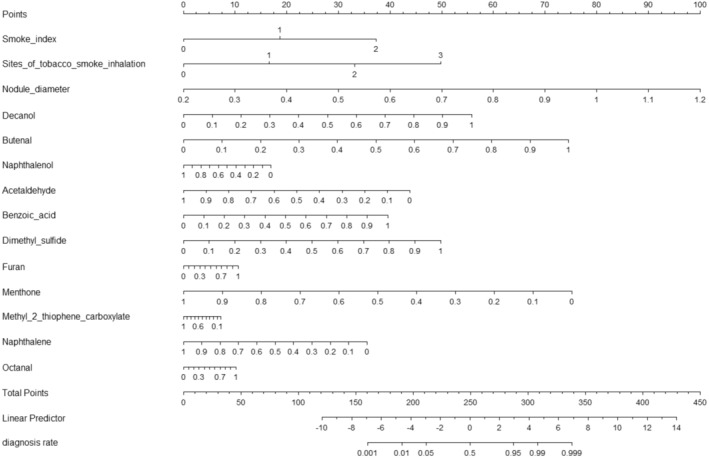
Nomogram of breathomics‐health examination‐lifestyle‐based predictive model to predict the probability of malignancy in individuals with pulmonary nodules. All exhaled VOCs were normalized using min‐max scaling.

The developed nomogram integrates multiple risk factors associated with pulmonary nodules, including the smoke index, site of tobacco smoke inhalation, nodule diameter, and 11 exhaled VOCs, with data provided in Table S4. Each predictor is assigned a specific point value that correlates directly with the potential risk of malignancy. For example, an individual with pulmonary nodules had a smoking index of 15 pack‐years (10–20 pack‐years) scoring 18 points, smoke inhaled into the mouth scoring 16 points, a nodule diameter of 0.5 cm scoring 30 points, and a concentration of 0.1 for all 11 VOCs (after normalization) scoring 5, 7, 16, 39, 4, 6, 1, 67, 7, 31, and 6, then the total score is approximately 253 (18 + 16 + 30 + 5 + 7 + 16 + 39 + 4 + 6 + 1 + 67 + 7 + 31 + 6). A total of 253 points corresponds to an estimated malignancy risk of approximately 55%. This percentage reflects the cumulative impact of all risk factors, as delineated by the nomogram (Figure 2).

## 4 Discussion

This study explores the potential value of breathomics in differentiating malignancy of pulmonary nodules and presents a comprehensive nomogram that integrates critical risk factors associated with the malignancy of pulmonary nodules, namely the smoke index, site of tobacco smoke inhalation, nodule diameter, and concentrations of exhaled VOCs. Integrating these VOCs with lifestyle and clinical factors significantly enhances the efficacy in assessing the malignancy of pulmonary nodules in asymptomatic individuals. By quantitatively combining these variables, our model offers a significant improvement over traditional methods that typically evaluate risk factors in isolation, thus enhancing predictive accuracy and supporting more targeted interventions. These findings highlight the potential of using exhaled VOCs in conjunction with other risk indicators to noninvasively assess malignant pulmonary nodules at an early stage, enabling timely interventions and potentially reducing the incidence of malignancy.

The detection rate of pulmonary nodules has indeed risen significantly in recent years, largely due to advances in imaging technologies such as LDCT scans [2, 5]. The presence of pulmonary nodules can significantly impact an individual's psychological well‐being due to the uncertainty and fear associated with potential cancer [39, 40, 41, 42, 43]. Therefore, developing new methods for assessing the malignancy of pulmonary nodules holds great promise for improving diagnostic accuracy and patient outcomes, of which, innovative methods were used to develop risk prediction models that integrate patient demographics, clinical history, and imaging features to provide a comprehensive assessment of the likelihood of malignancy. Particularly, considering the advantages of breath samples such as noninvasive, inexpensive, quick, and easy to collect [20, 24], the application of exhaled VOCs‐based assessment in clinical context seems promising, while more validations in diverse populations were needed.

Our study first explored a more comprehensive model by incorporating lifestyle factors, health examination data, and breathomics for assessing the malignancy of pulmonary nodules. Previous studies have predominantly focused on demographic characteristics, epidemiological data, and radiological signs to determine malignancy risk [44, 45, 46]. Factors such as older age, current or former smoking status, exposure to inhaled carcinogens (e.g., asbestos, radon, uranium), and the presence of emphysema or fibrosis, along with a family history of lung cancer, have been established as predictors of malignancy [44, 47, 48, 49]. Additionally, radiological features like large nodule diameter, spiculation, upper lobe location, and pleural indentation have been associated with higher malignancy risk [50, 51, 52, 53]. More recent research has incorporated plasma biomarkers, including IL‐6, IL‐10, IL‐1ra, C‐reactive protein (CRP), and low‐density lipoprotein cholesterol (LDL‐C), to differentiate malignant from benign pulmonary nodules [54, 55, 56, 57, 58, 59, 60], while others have explored urinary metabolites such as creatine riboside and N‐acetylneuraminic acid for diagnosis [61, 62, 63]. Our study extends this approach by demonstrating that specific VOCs in exhaled breath can also serve as noninvasive indicators for predicting nodule malignancy. This integration of multiple data sources, including novel breathomics, aligns with and builds upon existing research, aiming to enhance predictive accuracy and patient management.

The mechanism by which exhaled VOCs serve as diagnostic biomarkers in predicting the malignancy of pulmonary nodules involves the detection of metabolic changes associated with cancer. Clinical studies have shown that analyzing breath VOCs holds significant promise for the early screening of cancer and the detection of pulmonary diseases [64, 65, 66, 67, 68, 69]. Despite the potential, there is limited mechanistic research on the existence and metabolism of exhaled VOCs in asymptomatic patients. Studies on cancer patients have identified specific VOCs with potential diagnostic value [16, 70]. For instance, exhaled octanal, a product of endogenous lipid peroxidation, is associated with oxidative stress and can be elevated in the presence of malignancy [71]. It can also arise from smoking and dietary sources [72]. Since malignant cells typically exhibit heightened metabolic activity and oxidative stress [73, 74], elevated levels of octanal in exhaled breath might be observed in individuals at moderate risk for cancer [71]. In addition, dimethyl sulfide in breath is most often associated with halitosis [75], which is another compound of interest. Our study indicates an increase in dimethyl sulfide in the exhaled breath of individuals with moderate‐risk pulmonary nodules as compared to low‐risk controls, which is in support of previous findings conducted on lung cancer patients [76, 77, 78]. While Kischkel et al. reported that the concentration of dimethyl sulfide was lowest in lung cancer patients [79], they posited that their finding may be related to dental status rather than to cancer‐specific effects. Despite this, dimethyl sulfide has been identified as a key VOC breath biomarker for discrimination between lung cancer patients and healthy controls using decision tree classification [77]. Thus, by analyzing these VOCs, clinicians can enhance the diagnostic capabilities for predicting the malignancy of pulmonary nodules.

Interestingly, of the identified factors, the site of tobacco smoke inhalation was an independent predictor for moderate‐risk pulmonary nodules. This finding underscores the importance of understanding the environmental and contextual factors contributing to lung cancer risk, particularly in smokers. By examining where individuals are exposed to tobacco smoke—whether through direct smoking, second‐hand exposure, or occupational environments—we gain valuable insights into how specific inhalation patterns may exacerbate the risk of developing malignancies within pulmonary nodules. This finding aligns with existing literature, which indicates that as smoke moves deeper into the respiratory tract, more soluble gases are adsorbed, and particles are deposited in the airways and alveoli [80]. The inhalation of tobacco smoke often leads to the deposition of insoluble gases, such as carbon monoxide, that can reach the alveoli and diffuse across the alveolar‐capillary membrane [81]. These dosimetric considerations point to a heightened potential for lung injury among active smokers, reinforcing the importance of assessing inhalation patterns in evaluating the risk for pulmonary nodules.

Although the comprehensive breathomics‐health examination‐lifestyle‐based model demonstrated the best performance in predicting malignancy of pulmonary nodules, models based solely on lifestyle factors and lifestyle‐health examination data also showed acceptable performance. The breathomics component, integrating VOC analysis, significantly enhanced the predictive accuracy by offering insights into metabolic and oxidative changes not captured by other models [82]. However, even without breathomics, the lifestyle and health examination‐based models still provided valuable risk assessments. These models, incorporating factors such as smoking history, occupational exposures, and general health status, effectively stratified risk and supported early detection efforts. Thus, while breathomics adds a valuable dimension to diagnostic accuracy, lifestyle, and health examination data alone remain a robust alternative for risk prediction, particularly when comprehensive breath analysis is not feasible.

Our study integrated breathomics with lifestyle and health examination data, which enhances the predictive accuracy for malignancy in pulmonary nodules. Moreover, the use of VOC analysis provides a noninvasive, innovative approach to early detection, and the comprehensive model that combines breathomics with lifestyle and health factors offers a holistic view of risk, potentially improving diagnostic outcomes compared to traditional methods. However, several limitations of this study should be considered when interpreting these findings. First, variability in VOC profiles arises from external factors such as diet, environmental pollutants [83], and breath collection methods such as expiratory flow rate, breath hold, and inclusion of dead space [19], potentially undermining the reliability of breath‐based biomarkers. Second, using the peak area of extracted VOC as a substitute for concentration introduces bias in comparing exact VOC levels across groups. Third, many VOCs are not represented in the HMDB database, suggesting that potentially discriminating VOCs may have been overlooked. Fourth, reliance on historical data may not adequately reflect lifestyle or health condition changes over time, and there may be reporting bias in lifestyle variables (such as the site of tobacco inhalation) being self‐reported by participants although quality controls were conducted for the collected data. Furthermore, our study used the Mayo Clinic Model to identify benign and malignant pulmonary nodules, which, although a well‐acknowledged prediction model predicting malignancy risk, may not accurately reflect the actual disease state. Finally, the comparative group included only individuals with low‐risk and moderate‐risk pulmonary nodules, as no participants with high‐risk nodules were present. This limitation in sample size and diversity may restrict the applicability of the results across different populations. Future research should aim to address these limitations by enhancing sample diversity, incorporating longitudinal data, and refining VOC analysis techniques to improve the robustness and applicability of predictive models.

## 5 Conclusions

In conclusion, this study underscores the potential of integrating breathomics with lifestyle and health examination data to enhance the early detection and risk assessment of malignancy in pulmonary nodules. The comprehensive breathomics‐health examination‐lifestyle‐based model demonstrated superior performance, highlighting the value of VOC analysis in reflecting underlying pathological processes and oxidative stress. However, even models based solely on lifestyle and health examination data proved effective, emphasizing their continued relevance in risk prediction. Despite the promising results, the study's limitations, such as variability in VOC profiles and sample diversity, warrant further investigation. Continued research and refinement in breathomics and risk assessment models will be crucial in advancing early cancer detection and improving patient outcomes.

## Author Contributions

Weijuan Gong and Guangyu Lu had full access to all of the data in the study and took responsibility for the integrity of the data and the accuracy of the data analysis and also contributed to the concept and design of the manuscript. Guangyu Lu, Zhixia Su, and Weijuan Gong contributed to the drafting of the manuscript. Zhixia Su, Xiaoping Yu, Yuhang He, Taining Sha, Kai Yan, Yujian Tao, Hong Guo, Liting Liao, Yanyan Zhang, and Guotao Lu were involved in the statistical analysis. Weijuan Gong, Guangyu Lu, Xiaoping Yu, Yujian Tao, Hong Guo, Yanyan Zhang, and Guotao Lu contributed to administrative, technical, or material support. Weijuan Gong and Guangyu Lu were involved in supervision. All authors contributed to the acquisition, analysis, or interpretation of data and critical review of the manuscript for important intellectual content.

## Ethics Statement

The institutional review board of the Affiliated Hospital of Yangzhou University approved this study (2022‐YKL06‐SKJ005).

## Conflicts of Interest

The authors declare no conflicts of interest.

## Supporting information


**Figure S1:** Lasso regressions for candidate lifestyle factors and health examination data predictors. (a) LASSO regression coefficient path diagram; (b) LASSO regression cross‐validation curve.
**Figure S2:** Importance scores of variables incorporated into the breathomics‐health examination‐lifestyle‐based predictive model using RF algorithms.
**Figure S3:** AUCs comparison of five machine learning algorithms of (a) lifestyle‐based models, (b) health examination‐lifestyle‐based models, (c) breathomics‐health examination‐lifestyle‐based models.
**Figure S4:** Calibration curve of (a) lifestyle‐based LR model; (b) health examination‐lifestyle‐based LR model, and (c) breathomics‐health examination‐lifestyle‐based RF model.
**Figure S5:** Clinical decision curve of (a) lifestyle‐based LR model, (b) health examination‐lifestyle‐based LR model, and (c) breathomics‐health examination‐lifestyle‐based RF model.
Supporting Methods Section (A) Mayo Clinic model.
Section (B) Demographic and lifestyle factors of participants collected by using an adaptive questionnaire.
Section (C) Analytical instrumentation and parameters.
**Table S1.** Demographics, lifestyle factors, and health examination data of participants with pulmonary nodules in low‐ and moderate‐risk groups (*p* > 0.05).
**Table S2.** Exhaled VOCs of participants with pulmonary nodules in low‐ and moderate‐risk groups (*p* > 0.1).
**Table S3.** Twenty‐five candidate predictors for LASSO regression analysis.
**Table S4.** Exhaled VOCs used in the development of breathomics‐health examination‐lifestyle‐based predictive models.
**Table S5.** Variable screening in the logistic regression analysis to distinguish participants with pulmonary nodules in low‐risk group and moderate‐risk group.
**Table S6.** Definition of the variables incorporated into three predictive models.

## Data Availability

Data are available upon request with appropriate approvals.
