# Supplementary material for: Differentiating Pulmonary Nodule Malignancy Using Exhaled Volatile Organic Compounds: A Prospective Observational Study
Source: Cancer Med. 2025 Jan 7;14(1):e70545. doi: 10.1002/cam4.70545 (PMC11706237; doi:10.1002/cam4.70545)
Supplement: Supplementary file 1 — Figure S1: Lasso regressions for candidate lifestyle factors and health examination data predictors. (a) LASSO regression coefficient path diagram; (b) LASSO regression cross‐validation curve. Figure S2: Importance scores of variables incorporated into the breathomics‐health examination‐lifestyle‐based predictive model using RF algorithms. Figure S3: AUCs comparison of five machine learning algorithms of (a) lifestyle‐based models, (b) health examination‐lifestyle‐based models, (c) breathomics‐health examination‐lifestyle‐based models. Figure S4: Calibration curve of (a) lifestyle‐based LR model; (b) health examination‐lifestyle‐based LR model, and (c) breathomics‐health examination‐lifestyle‐based RF model. Figure S5: Clinical decision curve of (a) lifestyle‐based LR model, (b) health examination‐lifestyle‐based LR model, and (c) breathomics‐health examination‐lifestyle‐based RF model. Supporting Methods Section (A) Mayo Clinic model. Section (B) Demographic and lifestyle factors of participants collected by using an adaptive questionnaire. Section (C) Analytical instrumentation and parameters. Table S1. Demographics, lifestyle factors, and health examination data of participants with pulmonary nodules in low‐ and moderate‐risk groups (p > 0.05). Table S2. Exhaled VOCs of participants with pulmonary nodules in low‐ and moderate‐risk groups (p > 0.1). Table S3. Twenty‐five candidate predictors for LASSO regression analysis. Table S4. Exhaled VOCs used in the development of breathomics‐health examination‐lifestyle‐based predictive models. Table S5. Variable screening in the logistic regression analysis to distinguish participants with pulmonary nodules in low‐risk group and moderate‐risk group. Table S6. Definition of the variables incorporated into three predictive models. [file CAM4-14-e70545-s001.docx]

**Differentiating Pulmonary Nodule Malignancy Using Exhaled Volatile Organic Compounds: A Prospective Observational Study**

**Contents**

**Supplemental Methods**

**Section A)** Mayo Clinic model.

**Section B)** Demographic and lifestyle factors of participants collected by using an adaptive questionnaire.

**Section C)** Analytical instrumentation and parameters.

**Supplementary Tables**

**Supplementary Table S1.** Demographics, lifestyle factors, and health examination data of participants with pulmonary nodules in low- and moderate-risk groups ( *P*＞0.05 ).

**Supplementary Table S2.** Exhaled VOCs of participants with pulmonary nodules in low- and moderate-risk groups ( *P*＞0.1 ).

**Supplementary Table S3.** 25 candidate predictors for LASSO regression analysis.

**Supplementary Table S4.** Exhaled VOCs used in the development of breathomics-health examination-lifestyle based predictive models.

**Supplementary Table S5.** Variable screening in the logistic regression analysis to distinguish participants with PNs in low-risk group and moderate-risk group.

**Supplementary Table S6.** Definition of the variables incorporated into three predictive models.

**Supplementary Figures**

**Supplementary Figure S1:** Lasso regressions for candidate lifestyle factors and health examination data predictors. a) LASSO regression coefficient path diagram; b) LASSO regression cross-validation curve.

**Supplementary Figure S2:** Importance scores of variables incorporated into the breathomics-health examination-lifestyle based predictive model using RF algorithms.

**Supplementary Figure S3:** AUCs comparison of five machine learning algorithms of a) lifestyle based models, b) health examination-lifestyle based models, c) breathomics-health examination-lifestyle based models.

**Supplementary Figure S4:** Calibration curve of a) lifestyle based LR model; b) health examination-lifestyle based LR model, and c) breathomics-health examination-lifestyle based RF model.

**Supplementary Figure S5:** Clinical decision curve of a) lifestyle based LR model, b) health examination-lifestyle based LR model, and c) breathomics-health examination-lifestyle based RF model.

**Supplemental methods**

**Section A)** Mayo Clinic model.

This model is based on a logistic regression analysis and considers six independent predictors of malignancy: older age, history of smoking, history of an extrathoracic cancer more than 5 years before nodule detection, larger nodule diameter, upper lobe location, and spiculated margins [1]. The following equation was computed for the Mayo Clinic model. The pretest probability of a malignant SPN was ex/(1 + ex) for x = –6.8272 + (0.0391 × age) + (0.7917 × smoke) + (1.3388 × cancer) + (0.1274 × diameter) + 1.0407 × spiculation) + (0.7838 × upper lobe location), where e is the base of the natural logarithm, “age” indicates the patient’s age in years, “smoke” indicates smoking history in pack-years, “cancer” indicates history of an extrathoracic cancer 5 or more years before nodule identification (0, absent; 1, present in history), “diameter” indicates the measurement in millimeters of the largest nodule, “spiculation” indicates mention of nodule spiculation on any imaging test report (0, absent; 1, present on report), and “upper” indicates location of the nodule within the upper lobe of either lung (0, absent; 1, present in upper lobe).

**Section B)** Demographic characteristics and lifestyle factors of participants collected by using an adaptive questionnaire.

| **Questionnaire items** | **Features** |
| --- | --- |
| **General information** | health examination ID, gender, age, present address, ethnicity, marital status, education |
| **Smoking history** | Smoking (yes or no), quit smoking (yes or no), reasons for quitting smoking, smoking duration, the pack of cigarettes smoked per day, e-cigarette consumption (yes or no), sites of tobacco smoke inhalation |
| **Passive smoking & indoor air pollution** | exposure to secondhand smoke at home (yes or no), exposure duration to secondhand smoke at home, exposure to secondhand smoke in the workplace (yes or no), exposure duration to secondhand smoke in the workplace, cooking frequency every week, cooking fuel used, cooking oil used, cooking duration, house heating methods used, renovation and occupancy of housing |
| **Alcohol consumption** | alcohol intake frequency per week, drinking duration, types of alcohol consumption |
| **Tea drinking** | tea consumption (yes or no), drinking tea duration, types of tea consumption, frequency of the tea leaves changed per day, the concentration of tea, the heat of tea |
| **Coffee drinking** | coffee intake frequency per week, drinking coffee duration, the way coffee made, time for coffee |
| **Occupational exposure** | current occupation, types of occupation (day or nignt shift), history of occupational exposure (yes or no) |
| **Personal & family medical history** | have any symptoms (cough, .et al) (yes or no), history of any chronic diseases (yes or no), history of any pulmonary diseases (yes or no), Underwent a radiological examination in the past year (yes or no), duration of pulmonary nodules detected, family history of cancer |
| **Physical activities** | types of physical activity at work, working days per week, commuting mode, physical exercise (yes or no), types of physical exercise |
| **Drug use** | taking certain medications regularly (yes or no), drug name, dosage and frequency of drug use |
| **Sleeping** | average sleep time per day, snoring (yes or no), sleeplessness (yes or no), napping condition |
| **Diet** | regular meals (yes or no), frequency of cereal consumption per week, frequency of vegetables and fruits consumption per week, frequency of meat consumption per week, frequency of aquatic products consumption per week, frequency of milk and dairy products consumption per week, frequency of egg and their products consumption per week, frequency of beans and soy products consumption per week, frequency of nuts consumption per week, frequency of sweetmeat consumption per week, frequency of fried food consumption per week, frequency of somked food consumption per week |

**Section C)** Analytical instrumentation and parameters.

Thermal desorption tubes were stored at room temperature (20°C) before being desorbed onto a Unity 2 series thermal desorber coupled to a Pegasus 4D. The modulator was mounted in an Agilent 7890A gas chromatograph equipped with a secondary oven and a quad-jet dual stage modulator working with liquid nitrogen as cryofluid. The column set used was a combination of a Rxi-5Sil (30 m×0.25 mm i.d×0.25 μm df) in the first dimension (1D) and a BPX-50 (1.2 m×0.10 mm i.d×0.10 μm df) in the second dimension (2D). This column combination is classic but offers several advantages for non-targeted screening (e.g. structured separation). The use of this classic combination is also useful for study to study comparison since, it is the most common used combination. This column set was already successfully used in previous VOC mixtures untargeted analysis [2-4]. During the thermal desorption, samples were first purged with dry nitrogen during 6 min to remove water. Then, tubes were heated at 300°C during 10 min and VOCs samples were recollected on the general purposed cold trap (Tenax TA/Carbograph 1TD sorbent bed) at -10°C. Samples were injected in the system by heating of the cold trap at 300°C during 3 min. Helium was used as carrier gas with a constant flow rate of 1 mL/min. The main oven had an initial temperature of 25°C during 5 min and then increased until 300°C at a rate of 5°C/min. The temperature offset for the secondary oven was 5°C above the main oven. The modulation period (PM) was 2.4 s with a hot pulse duration set at 700 ms and a cooling time between stages of 1300 ms. The modulator temperature offset was 10°C above the temperature of the secondary GC oven. 70 eV electron ionization was used. The data acquisition rate was set at a frequency of 100 Hz for a mass range from 29 to 350 m/z.

**Reference**

[1] Swensen SJ, Silverstein MD, Ilstrup DM, Schleck CD, Edell ES. The probability of malignancy in solitary pulmonary nodules. Application to small radiologically indeterminate nodules. Arch Intern Med. 1997;157(8):849-855.

[2] Stadler S, Stefanuto PH, Brokl M, Forbes SL, Focant JF. Characterization of volatile organic compounds from human analogue decomposition using thermal desorption coupled to comprehensive two-dimensional gas chromatography-time-of-flight mass spectrometry. Anal Chem. 2013;85(2):998-1005.

[3] Dekeirsschieter J, Stefanuto PH, Brasseur C, Haubruge E, Focant JF. Enhanced characterization of the smell of death by comprehensive two-dimensional gas chromatography-time-of-flight mass spectrometry (GCxGC-TOF MS). PLoS One. 2012;7(6):e39005.

[4] Brasseur C, Dekeirsschieter J, Schotsmans EM, et al. Comprehensive two-dimensional gas chromatography-time-of-flight mass spectrometry for the forensic study of cadaveric volatile organic compounds released in soil by buried decaying pig carcasses. J Chromatogr A. 2012;1255:163-170.

**Supplementary Table S1.** Demographics, lifestyle factors, and health examination data of participants with pulmonary nodules in low- and moderate-risk groups ( *P*＞0.05 ).†

| **Variables** | **Total (n = 267)** | **Low-risk group**  **(n = 210)** | **Moderate-risk group**  **(n = 57)** | ***P*-value** |
| --- | --- | --- | --- | --- |
| Age, n (%) |  |  |  | 0.135 |
| 45-54 years | 152 (57) | 125 (60) | 27 (47) |  |
| 55-64 years | 115 (43) | 85 (40) | 30 (53) |  |
| Exposure to secondhand smoke at home, n (%) |  |  |  | 0.745 |
| Yes | 15 (6) | 13 (6) | 2 (4) |  |
| No | 252 (94) | 197 (94) | 55 (96) |  |
| Exposure duration to secondhand smoke at home (person-year) | 15/323 | 13/263 | 2/60 | 0.6016 |
| Exposure duration to secondhand smoke in the workplace (person-year) | 78/1342 | 53/932 | 25/410 | 0.7737 |
| Drinking duration (person-year) | 74/2027 | 46/1238 | 28/789 | 0.8480 |
| Drinking tea duration (person-year)* | 139/3182 | 100/2178 | 39/1004 | 0.3753 |
| Tea consumption per day（cup/day） | 139/1329 | 100/925 | 39/404 | 0.5484 |
| Coffee intake frequency, n (%) |  |  |  | 0.107 |
| Never | 230 (86) | 176 (84) | 54 (95) |  |
| ≤1 time/week | 11 (4) | 11 (5) | 0 (0) |  |
| 2-6 times/week | 9 (3) | 7 (3) | 2 (4) |  |
| Everyday | 17 (6) | 16 (8) | 1 (2) |  |
| Drinking coffee duration (person-year) | 37/674 | 34/614 | 3/60 | 0.8653 |
| Family history of cancer, n (%) |  |  |  | 0.071 |
| Yes | 64 (24) | 56 (27) | 8 (14) |  |
| No | 203 (76) | 154 (73) | 49 (86) |  |
| I-Bil ^¶^ | 10.6 (8.6, 13.25) | 10.6 (8.6, 13.75) | 10.6 (8, 13.1) | 0.666 |
| GPT/GOT ^¶^ | 0.95 (0.75, 1.21) | 0.96 (0.75, 1.22) | 0.9 (0.75, 1.21) | 0.644 |
| A/G ^§^ | 1.53 ± 0.18 | 1.52 ± 0.18 | 1.56 ± 0.18 | 0.195 |
| Globulin ^§^ | 30.01 ± 3.48 | 30.12 ± 3.35 | 29.63 ± 3.91 | 0.388 |
| Albumin ^§^ | 45.25 ± 1.9 | 45.24 ± 1.92 | 45.3 ± 1.84 | 0.815 |
| TP ^§^ | 75.26 ± 3.74 | 75.32 ± 3.68 | 75.05 ± 3.99 | 0.643 |
| DBIL ^¶^ | 3.7 (3.1, 4.5) | 3.7 (3.1, 4.6) | 3.7 (3, 4.4) | 0.42 |
| TBil ^¶^ | 14.5 (11.95, 17.5) | 14.4 (12.03, 17.58) | 14.5 (11.2, 17) | 0.69 |
| ALT ^¶^ | 21.4 (16.55, 29.65) | 21.25 (15.75, 28.6) | 24.25 (17.6, 34.9) | 0.079 |
| PV ^¶^ | 0.25 (0.22, 0.27) | 0.24 (0.22, 0.28) | 0.25 (0.22, 0.27) | 0.705 |
| MONO% ^¶^ | 6.2 (5.4, 7.3) | 6.1 (5.4, 7.3) | 6.55 (5.9, 7.3) | 0.116 |
| RDW ^¶^ | 12.7 (12.35, 13.1) | 12.74 (12.4, 13) | 12.7 (12.3, 13.2) | 0.669 |
| BASO% ^¶^ | 0.49 (0.3, 0.6) | 0.5 (0.3, 0.6) | 0.49 (0.3, 0.6) | 0.893 |
| EO% ^¶^ | 2 (1.3, 3.2) | 1.9 (1.3, 3.18) | 2.37 (1.4, 3.4) | 0.15 |
| NEUT% ^§^ | 53.42 ± 8.56 | 53.17 ± 8.87 | 54.32 ± 7.31 | 0.322 |
| LYMPH% ^§^ | 37.07 ± 8.36 | 37.45 ± 8.63 | 35.67 ± 7.16 | 0.116 |
| Basophil ^¶^ | 0.03 (0.02, 0.04) | 0.03 (0.02, 0.04) | 0.03 (0.02, 0.05) | 0.102 |
| Eosinophil ^¶^ | 0.12 (0.08, 0.21) | 0.11 (0.07, 0.2) | 0.14 (0.1, 0.24) | 0.072 |
| Lymphocytes ^¶^ | 2.23 (1.85, 2.68) | 2.21 (1.83, 2.68) | 2.29 (2, 2.69) | 0.314 |
| PDW ^¶^ | 16.1 (15.45, 16.4) | 16.1 (15.6, 16.4) | 16.1 (14.8, 16.4) | 0.773 |
| MCV ^¶^ | 91.6 (89.4, 94.2) | 91.54 (89.53, 94.18) | 92.1 (89.4, 94.4) | 0.377 |
| MCH ^¶^ | 30.3 (29.6, 31.3) | 30.2 (29.5, 31.2) | 30.44 (29.9, 31.4) | 0.173 |
| Platelet ^¶^ | 241 (209, 276.5) | 242 (210, 278.5) | 234 (206, 259) | 0.302 |
| MPV ^¶^ | 10.1 (9.4, 10.75) | 10.1 (9.4, 10.7) | 10.16 (9.4, 11) | 0.589 |
| MCHC ^¶^ | 330 (326.5, 334) | 330 (327, 334) | 331 (325, 336) | 0.331 |
| Fbg ^¶^ | 5.52 (5.14, 5.97) | 5.52 (5.14, 5.98) | 5.56 (5.17, 5.89) | 0.917 |
| AFP ^¶^ | 3.43 (2.64, 4.39) | 3.4 (2.59, 4.35) | 3.46 (2.85, 4.67) | 0.413 |
| LDL-C ^§^ | 2.82 ± 0.65 | 2.84 ± 0.67 | 2.78 ± 0.6 | 0.539 |
| TCH ^§^ | 5.32 ± 0.85 | 5.37 ± 0.86 | 5.16 ± 0.8 | 0.096 |
| CA199 ^¶^ | 9.53 (6.59, 13.15) | 9.53 (6.6, 13.3) | 9.53 (5.63, 12.2) | 0.477 |
| Urea ^¶^ | 4.96 (4.31, 5.78) | 4.94 (4.35, 5.78) | 5.06 (4.1, 5.76) | 0.446 |

Note:† The assessment of probability of malignancy in individuals with pulmonary nodules was reached according to the Mayo Clinic model. *: the number of people who drinking tea/the sum of years for drinking tea of each person, ^§:^ mean±standard deviation, ^¶^: median (P25, P75); Abbreviations: I-Bil: Indirect Bilirubin; GPT/GOT: glutamic pyruvic transaminase/glutamic oxaloacetic transaminase; A/G: Albumin/Globulin; TP: Total Protein; TBil: Total bilirubin; ALT: Alanine aminotransferase; PV: platelet volume; MONO%: Monocyte ratio; RDW: Red blood cell distribution width; BASO%: Basophil ratio; EO%: Eosinophil ratio; NEUT%: Neutrophil ratio; LYMPH%: Lymphocyte ratio; PDW: Platelet distribution width; MCV: Mean corpuscular volume; MCH: Mean corpuscular hemoglobin; MPV: mean platelet volume; NE: Neutrophilicgranulocyte; MCHC: Mean corpuscular hemoglobin concentration; Fbg: Fasting blood glucose; AFP:Alpha-fetoprotein; LDL-C: Low-Density Lipoprotein Cholesterol; TCH: Total cholesterol; CA199: Carbohydrate antigen199.

**Supplementary Table S2.** Exhaled VOCs of participants with pulmonary nodules in low- and moderate-risk groups ( *P*＞0.1 ).†

| **Exhaled VOCs** | **Total (n = 267)** | **Low-risk group**  **(n = 210)** | **Moderate-risk group**  **(n = 57)** | ***P*-value** |
| --- | --- | --- | --- | --- |
| (+)-3-Carene ^‡^ | 21043068.09 (5822460.11, 57197557.32) | 20972557.13 (6206585.42, 57388362.44) | 21098604.47 (3406141.11, 56625141.95) | 0.897 |
| 10-Undecen-1-ol ^‡^ | 1979339.85 (516672.2, 4849491.06) | 2028897.4 (466330.47, 4778649.01) | 1662592.96 (528072.35, 5147028.2) | 0.865 |
| 1-Dodecanol ^§^ | 0 (0, 752240.03796) | 0 (0, 737491.79682) | 0 (0, 934492.05711) | 0.478 |
| 1-Dodecene ^‡^ | 0 (0, 2033424.58) | 0 (0, 1876786.56) | 0 (0, 2288469.31) | 0.736 |
| 1-Hexadecanol ^‡^ | 637092.57 (201493.78, 1458100.75) | 677699.52 (250472.52, 1436978.32) | 439388.16 (0, 1992086.73) | 0.443 |
| 1-Naphthalenol ^§^ | 0 (0, 156361.98874) | 0 (0, 156323.77603) | 0 (0, 190945.54437) | 0.506 |
| 1-Nonanol ^§^ | 0 (0, 494382.89658) | 0 (0, 485928.95029) | 0 (0, 596557.73589) | 0.764 |
| 1-Nonen-3-ol ^§^ | 0 (0, 2780294.1214) | 0 (0, 3376582.40385) | 0 (0, 1552228.3578) | 0.761 |
| 1-Nonene ^‡^ | 0 (0, 518594.29) | 0 (0, 555215.95) | 0 (0, 287524.5) | 0.341 |
| 1-Octanol ^‡^ | 0 (0, 964118.35) | 0 (0, 742768.86) | 0 (0, 1497999.8) | 0.525 |
| 1-Octen-3-ol ^§^ | 0 (0, 4211004.3492) | 0 (0, 3878943.87425) | 0 (0, 5949087.7759) | 0.501 |
| 1-Octene ^‡^ | 0 (0, 817111.53) | 0 (0, 532259.17) | 0 (0, 2513588.16) | 0.52 |
| 1-Pentanol ^§^ | 0 (0, 2011718.0506) | 0 (0, 2128865.8936) | 0 (0, 1239779.5293) | 0.246 |
| 1-Tetradecene ^§^ | 0 (0, 452245.65818) | 0 (0, 451561.56776) | 0 (0, 798548.77704) | 0.697 |
| 1-Tridecene ^‡^ | 468228.32 (0, 1558771.03) | 491854.97 (0, 1526264.52) | 228388.11 (0, 1569606.53) | 0.539 |
| 1-Undecanol ^‡^ | 0 (0, 584737.79) | 0 (0, 634044.8) | 0 (0, 511625.97) | 0.775 |
| 1-Undecene ^‡^ | 1699527.26 (0, 7554211.3) | 1712031.54 (0, 7309140.67) | 1687022.98 (0, 7635901.52) | 0.959 |
| 2-Butanone ^§^ | 0 (0, 1292682.0806) | 0 (0, 993840.270595) | 0 (0, 2416489.1224) | 0.507 |
| 2-Propenenitrile ^§^ | 0 (0, 689049.58496) | 0 (0, 717320.317575) | 0 (0, 689131.23042) | 0.643 |
| 2-Propenoic acid ^§^ | 0 (0, 792898.88722) | 0 (0, 859445.60199) | 0 (0, 705034.28547) | 0.556 |
| 2-Thiophenecarboxaldehyde ^§^ | 0 (0, 196597.92758) | 0 (0, 208902.80089) | 0 (0, 19377.22931) | 0.589 |
| 3-Carene ^‡^ | 0 (0, 1002103.19) | 0 (0, 989450.76) | 200921.18 (0, 1054922.04) | 0.458 |
| 3-Octanone ^§^ | 0 (0, 1020248.0566) | 0 (0, 1122993.49665) | 0 (0, 961646.55827) | 0.332 |
| 6-Tridecene ^‡^ | 0 (0, 222177.97) | 0 (0, 0) | 0 (0, 508516.96) | 0.127 |
| Acetamide ^§^ | 0 (0, 524809.23948) | 0 (0, 470099.101105) | 0 (0, 771332.70023) | 0.826 |
| Acetic acid ^‡^ | 7154971.04 (2559210.06, 15685245.14) | 6825644.53 (0, 15321903.88) | 8216344.5 (3894937.75, 17873499.97) | 0.255 |
| Acetone ^‡^ | 154452817.95 (113425775.65, 211094931.18) | 153282822 (114809857.3, 212139697.6) | 159249531.2 (113001035.1, 195673534.5) | 0.875 |
| Acetonitrile ^‡^ | 20147803.69 (9743187.33, 46917547.42) | 20566567.45 (10033792.4, 47363784.64) | 18777109.71 (8589112.25, 45703377.7) | 0.763 |
| Acetophenone ^‡^ | 4210381.15 (2605606.69, 7149031.73) | 4107411.43 (2596445.26, 6831861.7) | 4386318 (2650053.95, 7617088.68) | 0.409 |
| Alpha-Methylstyrene ^‡^ | 2845645.71 (1478826.9, 5533380.09) | 2804627.6 (1513542.27, 5700826.43) | 2909202.29 (1421470.53, 5389249) | 0.946 |
| alpha-Phellandrene ^‡^ | 0 (0, 1800891.25) | 0 (0, 2370493.72) | 0 (0, 414864.92) | 0.595 |
| alpha-Terpineol ^‡^ | 2729582.02 (1424163.36, 4576253.72) | 2671020.5 (1558483.82, 4639869.3) | 2844049.28 (1214726.96, 4018913.24) | 0.751 |
| Anethole ^‡^ | 0 (0, 178250.9) | 0 (0, 202066.65) | 0 (0, 0) | 0.161 |
| Argon ^‡^ | 10031483.73 (9528002.34, 10950277.03) | 10054431.29 (9530619.17, 10988305.46) | 9984296.44 (9487635.15, 10904093.57) | 0.649 |
| Benzaldehyde ^‡^ | 20010296.38 (11747675.66, 37656602.57) | 19396722.78 (11537445.92, 38203980.08) | 23785291.87 (12684608.4, 34489959.53) | 0.48 |
| Benzene ^‡^ | 87595027.34 (76889553.48, 102348067.1) | 87593117.81 (76436600.98, 102297293.5) | 88849256.59 (77954343.78, 107996352.4) | 0.786 |
| Benzofuran ^‡^ | 162065.41 (0, 1334336.85) | 0 (0, 1328274.2) | 496174.56 (0, 1801446.44) | 0.601 |
| Benzonitrile ^‡^ | 2431640.59 (907923.46, 6555171.41) | 2422539.58 (850193.5, 6729550.99) | 2820297.68 (1276839.61, 6333461.69) | 0.25 |
| Benzophenone ^‡^ | 556442.71 (318247.09, 1029567.56) | 548844.38 (310195.16, 1044991.39) | 685659.51 (379291.55, 906716.12) | 0.409 |
| Benzothiazole ^§^ | 0 (0, 147706.5128) | 0 (0, 141277.024425) | 0 (0, 176127.4228) | 0.172 |
| Benzyl alcohol ^‡^ | 7008564.45 (3657991.43, 12972276.64) | 7153508.72 (3823387.19, 12973401.17) | 6927221.8 (3252380.33, 12967892.31) | 0.728 |
| beta-Phellandrene ^‡^ | 0 (0, 351616.76) | 0 (0, 223089.38) | 0 (0, 372345.41) | 0.509 |
| Beta-Pinene ^‡^ | 2618882.6 (0, 23015040.42) | 2399040.35 (0, 22725787.14) | 3115170.66 (413306.82, 23162592.61) | 0.512 |
| Biphenyl ^‡^ | 554983.15 (426929.92, 697176.61) | 545910.48 (426473.22, 680425.34) | 590551.47 (429508.56, 721476.52) | 0.458 |
| Butanal ^§^ | 0 (0, 3328357.1598) | 0 (0, 10137223.04665) | 0 (0,1396267.9357) | 0.803 |
| Butanoic acid ^‡^ | 0 (0, 0) | 0 (0, 0) | 0 (0, 209218.01) | 0.265 |
| Butylated Hydroxytoluene^§^ | 0 (0, 217277.57548) | 0 (0, 222716.342985) | 0 (0, 221041.35109) | 0.776 |
| Camphene ^‡^ | 1536311.82 (747597.13, 6387616.16) | 1514811.56 (754806.61, 5791512.07) | 1557812.07 (703753.09, 8548214.54) | 0.876 |
| Carbon dioxide ^§^ | 0 (0, 62618.99706) | 0 (0, 0) | 0 (0, 2049573.804) | 0.738 |
| Carbon disulfide ^‡^ | 0 (0, 6048159.13) | 0 (0, 6169017.27) | 804196.39 (0, 5596335.98) | 0.947 |
| Carbon Tetrachloride ^‡^ | 1072283.53 (0, 1728476.78) | 1100128.54 (0, 1692925.47) | 843998.14 (0, 1776678.78) | 0.606 |
| Carveol ^‡^ | 801161.32 (0, 2025499.96) | 796127.67 (0, 2117704.27) | 819383.73 (0, 1749004.66) | 0.818 |
| Carvone ^§^ | 0 (0, 2320317.7952) | 0 (0, 2276651.94095) | 0 (0, 3023878.3404) | 0.196 |
| Caryophyllene ^‡^ | 0 (0, 3052326.08) | 0 (0, 3099493.04) | 0 (0, 2838023.86) | 0.917 |
| Cedrene ^‡^ | 498534.8 (0, 1315622.49) | 499381.93 (0, 1287279.52) | 497687.66 (212984.73, 1458792.44) | 0.718 |
| cis-3-Decene ^‡^ | 1540808.15 (0, 10316900.83) | 1896502.23 (0, 10351532.67) | 0 (0, 9101485.81) | 0.238 |
| cis-Hept-4-enol ^§^ | 0 (0, 395025.58696) | 0 (0,388412.72482) | 0 (0, 448541.02367) | 0.44 |
| Cyclohexane ^‡^ | 6625708.47 (4968629.65, 9907181.37) | 6645724.05 (5047883.15, 9971947.76) | 6551638.34 (4565788.57, 8853839.4) | 0.398 |
| Cyclotetradecane ^§^ | 0 (0, 1731122.7626) | 0 (0, 2145469.34895) | 0 (0, 69369.6792) | 0.169 |
| Decanal ^‡^ | 0 (0, 291450.69) | 0 (0, 0) | 0 (0, 951792.35) | 0.211 |
| Decane ^‡^ | 266423.57 (0, 1655313.48) | 336595.06 (0, 1751089.4) | 0 (0, 1132801.17) | 0.212 |
| Dibutyl phthalate ^‡^ | 436017.47 (0, 1099389.38) | 447769.98 (0, 1129215.1) | 0 (0, 1012059.64) | 0.747 |
| Diethyl Phthalate ^‡^ | 5090362.86 (2840228.18, 12050449.99) | 4888366.92 (2804866.11, 11009344.76) | 6129619.21 (3219608.46, 13386997.89) | 0.18 |
| Dimethyl sulfone ^‡^ | 0 (0, 398655.94) | 0 (0, 402493.05) | 0 (0, 387258.1) | 0.737 |
| Dimethyl trisulfide ^§^ | 0 (0, 240695.67662) | 0 (0, 257211.948125) | 0 (0, 242410.75472) | 0.951 |
| Diphenyl ether ^§^ | 0 (0, 6871158.289) | 0 (0, 6049797.2418) | 0 (0, 8053824.7336) | 0.378 |
| D-Limonene ^‡^ | 361811.4 (0, 1636468.51) | 346215.94 (0, 1577263.1) | 432347.15 (0, 1937766.09) | 0.555 |
| Dodecanal ^‡^ | 0 (0, 559924.44) | 0 (0, 585466.6) | 0 (0, 431223.96) | 0.587 |
| Dodecane ^‡^ | 4172955.82 (936936.32, 9000199.66) | 3996843.03 (846418.85, 8677598.09) | 5683469.06 (1220280.09, 12072356.5) | 0.166 |
| Dodecanoic acid ^‡^ | 691226.88 (0, 1308280.67) | 694794.22 (0, 1332244.12) | 673156.34 (0, 1250599.39) | 0.815 |
| Estragole ^‡^ | 182671.99 (0, 164696545.25) | 223297.3 (0, 165369740.9) | 0 (0, 130929031.7) | 0.719 |
| Ethyl Acetate ^‡^ | 24094810.18 (15997840.66, 34110972.48) | 24101857.84 (15962776.94, 33493888.57) | 24087762.51 (16148649.53, 37248391.98) | 0.926 |
| Ethylbenzene ^‡^ | 11220466.72 (8500867.06, 18386090.96) | 11522255.92 (8592604.25, 18458672.18) | 10642349.78 (8082546.89, 17261068.86) | 0.547 |
| Furfural ^‡^ | 0 (0, 442763.5) | 0 (0, 445201.52) | 164322.4 (0, 403236.74) | 0.368 |
| Glutaraldehyde ^§^ | 0 (0, 13742237.73) | 0 (0, 10342492.9305) | 0 (0, 19321264.351) | 0.315 |
| Heptadecane ^‡^ | 1638949.07 (0, 4904307.91) | 1597186.19 (0, 4962098.71) | 2558934.01 (0, 4871832.64) | 0.877 |
| Heptane ^‡^ | 12244015.34 (8110954.83, 17791147.8) | 12561257.86 (8582387.51, 17844809.08) | 10924155.85 (6935458.99, 17630163.96) | 0.21 |
| Hexadecane ^‡^ | 22658007.84 (6009294.12, 40467762.51) | 22282755.26 (5985513.38, 36296744.2) | 25864889.97 (6405195.86, 42744638.34) | 0.65 |
| Hexanoic acid ^‡^ | 0 (0, 0) | 0 (0, 168537.5) | 0 (0, 0) | 0.313 |
| Indane ^‡^ | 0 (0, 271046.25) | 0 (0, 255061.14) | 0 (0, 284470.06) | 0.696 |
| Isopropyl Alcohol ^‡^ | 41181191.06 (21899888.37, 72758932.64) | 43103458.62 (23980067.88, 79363463.73) | 36119840.1 (19015661.98, 62728931.46) | 0.135 |
| Isopulegol acetate ^§^ | 0 (0, 3497447.2326) | 0 (0, 7267876.22605) | 0 (0, 1727802.2883) | 0.207 |
| Limonene ^‡^ | 1691102.79 (0, 6809697.28) | 1576481.98 (0, 6100890.82) | 2249755.98 (225727.64, 13455269.18) | 0.132 |
| Mesitylene ^‡^ | 1307525.74 (748665.92, 1987683.32) | 1309209.9 (804184.9, 1982520.79) | 1269021.63 (640545.1, 1989404.16) | 0.756 |
| Methacrolein ^‡^ | 1185109.81 (504761.69, 2231879.3) | 1177750.59 (619489.33, 2044619.81) | 1283928.66 (456111.78, 2616252.32) | 0.529 |
| Methenamine ^§^ | 0 (0, 623110.1241) | 0 (0, 693340.933195) | 0 (0, 442360.93068) | 0.188 |
| Methyl methacrylate ^§^ | 0 (0, 2692076.2306) | 0 (0, 1927692.57935) | 0 (0, 4599039.7752) | 0.428 |
| Methyl salicylate ^§^ | 0 (0, 13560.07185) | 0 (0, 0) | 0 (0, 13560.07185) | 0.908 |
| Methylene chloride ^‡^ | 5775325.12 (2317209.17, 11694981.77) | 6060499.42 (2753614.65, 11569633.97) | 3601552.33 (1759338.67, 13275638.39) | 0.112 |
| Myristoleic acid ^§^ | 0 (0, 67866.04611) | 0 (0, 0) | 0 (0, 67866.04611) | 0.783 |
| n-Decanoic acid ^§^ | 0 (0, 349930.56232) | 0 (0, 381553.536585) | 0 (0, 350989.53582) | 0.787 |
| n-Hexadecanoic acid ^‡^ | 4512039.39 (2885641.97, 6932123.1) | 4599051.07 (3129470.04, 6802076.46) | 4242270.8 (1609004.82, 7531059.25) | 0.688 |
| n-Hexane ^‡^ | 31014861.68 (22135675.38, 59375149.72) | 31232128.95 (22591674.85, 64602953) | 30391409.62 (20548785.63, 43101487.54) | 0.204 |
| Nonanal ^‡^ | 0 (0, 1569734.41) | 0 (0, 1320500.57) | 0 (0, 1901699.44) | 0.824 |
| Nonane ^‡^ | 527502.51 (171334.38, 1128968.59) | 506428.13 (160483.04, 1163408.9) | 597916.93 (233753.26, 1049754.87) | 0.711 |
| Nonanoic acid ^‡^ | 306357.26 (0, 505069.61) | 304260.72 (0, 495424.09) | 312514.01 (0, 548665.23) | 0.801 |
| n-Propyl acetate ^§^ | 0 (0, 434261.79278) | 0 (0, 471973.90847) | 0 (0, 135273.9164) | 0.398 |
| Octadecanoic acid ^‡^ | 0 (0, 1466774.82) | 0 (0, 1491343.55) | 0 (0, 954849.48) | 0.256 |
| Octane ^§^ | 0 (0, 1052135.737) | 0 (0, 974937.696535) | 0 (0, 1322450.4326) | 0.355 |
| Octanoic acid ^§^ | 0 (0, 276090.96792) | 0 (0, 276041.82519) | 0 (0, 355356.15437) | 0.203 |
| o-Cymene ^‡^ | 4242655.22 (1267648.56, 22957414.41) | 4215538.56 (1262126.48, 23495527.38) | 4269771.88 (1381058.22, 21565649.29) | 0.969 |
| Oleic Acid ^§^ | 0 (0, 2534228.2528) | 0 (0, 50777596.027) | 0 (0, 66242092.843) | 0.639 |
| o-Xylene ^§^ | 0 (0, 364643.00598) | 0 (0, 353358.25627) | 0 (0, 403653.34131) | 0.335 |
| p-Cymene ^‡^ | 0 (0, 390301.01) | 0 (0, 450166.86) | 0 (0, 271163.1) | 0.104 |
| Pentadecane ^‡^ | 74992607.06 (38010691.8, 125174090.95) | 73022221.61 (37897255.82, 124383214.6) | 78519380.68 (40273745.71, 139867631.1) | 0.643 |
| Pentanal ^§^ | 0 (0, 7665680.9826) | 0 (0, 8568612.34255) | 0 (0, 7024987.0015) | 0.659 |
| Pentane ^‡^ | 34398700.31 (19272143.75, 77560790.2) | 36402958.41 (19314629.02, 81736478.78) | 30461963.67 (19257981.99, 56783873.29) | 0.258 |
| Pentanoic acid ^‡^ | 0 (0, 452036.74) | 0 (0, 476990.63) | 0 (0, 418172.5) | 0.593 |
| Phenol ^‡^ | 3921123.97 (2705644.84, 7448510.7) | 3964723.82 (2720586.63, 7517245.66) | 3832999.36 (2700664.24, 7280527.53) | 0.954 |
| Phenylglyoxal ^§^ | 0 (0, 116323.000672) | 0 (0, 24923.054556) | 0 (0, 6567570.5672) | 0.471 |
| Propanoic acid ^‡^ | 0 (0, 0) | 0 (0, 0) | 0 (0, 480565.65) | 0.438 |
| p-Xylene ^§^ | 0 (0, 1116105.0958) | 0 (0, 1157485.96735) | 0 (0, 848886.69138) | 0.577 |
| Pyridine ^§^ | 0 (0, 1514355.3516) | 0 (0, 1574671.41875) | 0 (0, 1282001.8632) | 0.552 |
| Pyrrole ^§^ | 0 (0, 546292.15128) | 0 (0, 579688.70143) | 0 (0, 432760.97296) | 0.553 |
| Styrene ^‡^ | 6550899.12 (4146847.17, 14253858.76) | 6896024.99 (4124028.26, 14431354.56) | 6037356.61 (4215303.88, 13951355.96) | 0.864 |
| Sulfur dioxide ^‡^ | 8505038.93 (6305120.8, 13998242.88) | 8299129.43 (6249664.77, 13069549.1) | 9542017.19 (6573518.03, 15085004.14) | 0.138 |
| Tetrachloroethylene ^‡^ | 522650.85 (294504.97, 896952.35) | 550465.64 (311977.37, 901704.08) | 470464.27 (263739.72, 738685.52) | 0.226 |
| Tetradecane ^‡^ | 58725702.55 (26040631.13, 139137862.32) | 56347318.7 (25639279.74, 136550957) | 67424447.28 (28856207.7, 163699085.6) | 0.474 |
| Tetrahydrofuran ^§^ | 0 (0, 1274904.8866) | 0 (0, 1271313.56845) | 0 (0, 1370025.9022) | 0.454 |
| Thiophene ^‡^ | 0 (0, 970216.05) | 0 (0, 948170.71) | 0 (0, 1051566.42) | 0.636 |
| Thymol ^§^ | 0 (0, 287707.5216) | 0 (0, 311547.612575) | 0 (0, 282745.36457) | 0.462 |
| Toluene ^‡^ | 31923330.5 (19495279.34, 49275683.11) | 32946753.78 (19442443.6, 49540787.18) | 27245673.99 (19965060.61, 47842754.07) | 0.638 |
| Trichloroethylene ^‡^ | 32675.21 (0, 256581.53) | 74458.61 (0, 269408.8) | 0 (0, 220212.01) | 0.508 |
| Trichloromethane ^‡^ | 5040854.07 (3536407.51, 8073327.52) | 5197325.15 (3643171.14, 7896877.6) | 4464711.55 (3504835.24, 8089848.65) | 0.206 |
| Tridecane ^‡^ | 1460445.49 (184546.21, 3089890.24) | 1605209.28 (209652.22, 3239651.86) | 1075950.72 (152432.07, 2721025) | 0.499 |
| Undecanal ^§^ | 0 (0, 478215.10626) | 0 (0, 396278.44281) | 0 (0, 2107153.0079) | 0.618 |
| Undecane ^‡^ | 769792.55 (0, 2834274.39) | 708246.29 (0, 2689398.6) | 1039186.68 (0, 3225402.48) | 0.364 |

Note: Abbreviations: VOCs: Volatile organic compounds. †: The assessment of probability of malignancy in individuals with pulmonary nodules was reached according to the Mayo Clinic model. Skewed distributed quantitative data were presented as median (percentile range) and compared between groups using the Mann-Whitney U test. ^‡:^ median (P25, P75), ^§^: median (P5, P95); ^¶^: median (P1, P100).

**Supplementary Table S3**. 25 candidate predictors for LASSO regression analysis.

| **Characteristics** | **Categories and assignments** | **Unit of concentration** |
| --- | --- | --- |
| **A) Epidemiological data** |  |  |
| Age | 0=45-54, 1=55-64 | years |
| Smoke index | 0=＜10, 1=10-20, 2=≥20 | pack-year |
| Sites of tobacco smoke inhalation | 0=Never smoke, 1=Inhaled into mouth, 2=Inhaled into throat, 3=Inhaled into lung | - |
| Exposure to secondhand smoke from coworkers | 0=No, 1=Yes | - |
| Alcohol intake frequency | 0=Never, 1=≤1 time/week, 2=2-6 times/week, 3=Every day, 4=Abstinent from alcohol | - |
| Tea intake frequency | 0=No, 1=Yes | - |
| Family history of cancer | 0=No, 1=Yes | - |
| Working hours per day | Continuous variable | hours |
| **B) Health examination information** |  |  |
| Nodule diameter | Continuous variable | cm |
| GGT | Continuous variable | U/L |
| AST | Continuous variable | U/L |
| ALT | Continuous variable | U/L |
| Eosinophil | Continuous variable | *10^9/L |
| Monocyte | Continuous variable | *10^9/L |
| HCT | Continuous variable | % |
| RBC | Continuous variable | *10^12/L |
| Hemoglobin | Continuous variable | g/L |
| WBC | Continuous variable | *10^12/L |
| NE | Continuous variable | *10^9/L |
| CEA | Continuous variable | ng/ml |
| HDL-C | Continuous variable | mmol/L |
| TG | Continuous variable | mmol/L |
| TC | Continuous variable | mmol/L |
| AU | Continuous variable | umol/L |
| CR | Continuous variable | umol/L |

Abbreviations: GGT: Gamma Glutamyl Transferase; AST: Aspartate Transaminase; ALT: Alanine aminotransferase; HCT: Hematocrit; RBC: Red blood cells; WBC: White blood cell; NE: Neutrophilicgranulocyte; CEA: Carcinoembryonic antigen; HDL-C: High density lipoprotein cholesterol; TG: Triglyceride; TC: Total Cholesterol; AU: Aric acid; CR: Creatinine.

**Supplementary Table S4.** Exhaled VOCs used in the development of breathomics-health examination-lifestyle based predictive models.

| **Exhaled VOCs** | **Chemical family** | **CAS number** |
| --- | --- | --- |
| 2-Butenal | Aldehydes | 4170-30-3 |
| Acetaldehyde | Aldehydes | 75-07-0 |
| Octanal | Aldehydes | 124-13-0 |
| 2-Naphthalenol | Aromatic compounds | 135-19-3 |
| Naphthalene | Aromatic compounds | 91-20-3 |
| Benzoic acid | Acids | 117500-35-3 |
| 1-Decanol | Alcohols | 112-30-1 |
| Dimethyl sulfide | Sulfocompounds | 75-18-3 |
| Furan | Oxygenheterocycles | 110-00-9 |
| Methyl-2-thiophene carboxylate | Esters | 90179-20-7 |
| l-Menthone | Others | 14073-97-3 |

Abbreviation: PNs: Pulmonary nodules; VOCs: Volatile Organic compounds; CAS: Chemical abstracts service

**Supplementary Table S5.** Variable screening in the logistic regression analysis to distinguish participants with PNs in low-risk group and moderate-risk group.

| **Variable** | **Odds ratio** | **lower** | **Upper** | ***P-*value** |
| --- | --- | --- | --- | --- |
| Smoke index | 23.08 | 5.18 | 102.9 | <0.001 |
| Sites of tobacco smoke inhalation | 19.4 | 4.4 | 85.5 | <0.001 |
| Nodule diameter | 578.6 | 47.5 | 7045.8 | <0.001 |
| Constant | 0.01 | - | - | 0.946 |

**Supplementary Table S6.** Definition of the variables incorporated into three predictive models.

| **Characteristics** | **Definition** | **Categories and assignments** | **Unit of concentration** |
| --- | --- | --- | --- |
| **A) Epidemiological data** |  |  |  |
| Smoke index | The smoke index, calculated by multiplying the packs of cigarettes smoked per year by the number of years of smoking. | 0=＜10, 1=10-20, 2=≥20 | pack-year |
| Sites of tobacco smoke inhalation | The site of tobacco smoke inhalation is defined as whether the smoke is inhaled into the mouth, throat or deep into the lungs when smoking. | 0= Never smoke, 1=Inhaled into mouth, 2=Inhaled into throat, 3=Inhaled into lung | - |
| **B) Health examination information** |  |  |  |
| Nodule diameter | The maximum diameter of pulmonary nodules on CT images. | Continuous variable | cm |
| **C) Exhaled volatile organic compounds** |  |  |  |
| 1-Decanol | The concentration 1-Decanol was presented by the peak area indefied by TD-GC×GC-TOF MS. | Continuous variable | - |
| 2-Butenal | The concentration 2-Butenal was presented by the peak area indefied by TD-GC×GC-TOF MS. | Continuous variable | - |
| 2-Naphthalenol | The concentration 2-Naphthalenol was presented by the peak area indefied by TD-GC×GC-TOF MS. | Continuous variable | - |
| Acetaldehyde | The concentration Acetaldehyde was presented by the peak area indefied by TD-GC×GC-TOF MS. | Continuous variable | - |
| Benzoic acid | The concentration Benzoic acid was presented by the peak area indefied by TD-GC×GC-TOF MS. | Continuous variable | - |
| Dimethyl sulfide | The concentration Dimethyl sulfide was presented by the peak area indefied by TD-GC×GC-TOF MS. | Continuous variable | - |
| Furan | The concentration Furan was presented by the peak area indefied by TD-GC×GC-TOF MS. | Continuous variable | - |
| l-Menthone | The concentration l-Menthone was presented by the peak area indefied by TD-GC×GC-TOF MS. | Continuous variable | - |
| Methyl-2-thiophene carboxylate | The concentration Methyl-2-thiophene carboxylate was presented by the peak area indefied by TD-GC×GC-TOF MS. | Continuous variable | - |
| Naphthalene | The concentration Naphthalene was presented by the peak area indefied by TD-GC×GC-TOF MS. | Continuous variable | - |
| Octanal | The concentration Octanal was presented by the peak area indefied by TD-GC×GC-TOF MS. | Continuous variable | - |

Abbreviation: CT: computed tomography; TD-GC×GC-TOF MS: Comprehensive two-dimensional gas chromatography coupled to time-of-flight mass spectrometry.


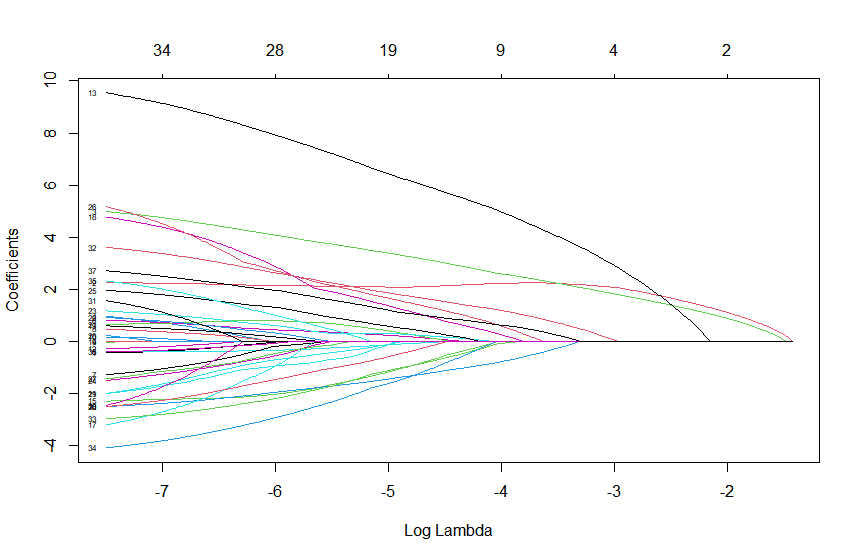

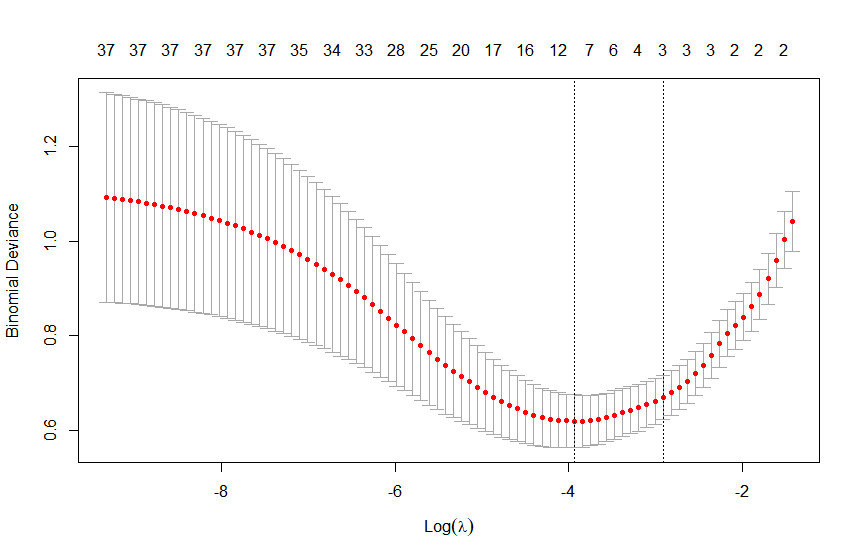


a)

b)

**Supplementary Figure S1. Lasso regressions for candidate lifestyle factors and health examination data predictors.** Note: a) LASSO regression coefficient path diagram; b) LASSO regression cross-validation curve. Three predictors with nonzero coefficients were selected by LASSO regression (screening lambda by 10-fold cross-validation, based on lambda. 1se, i.e., the maximum lambda corresponding to an error mean within one standard deviation of the minimum): Smoke index, sites of tobacco smoke inhalation, and nodule diameter.


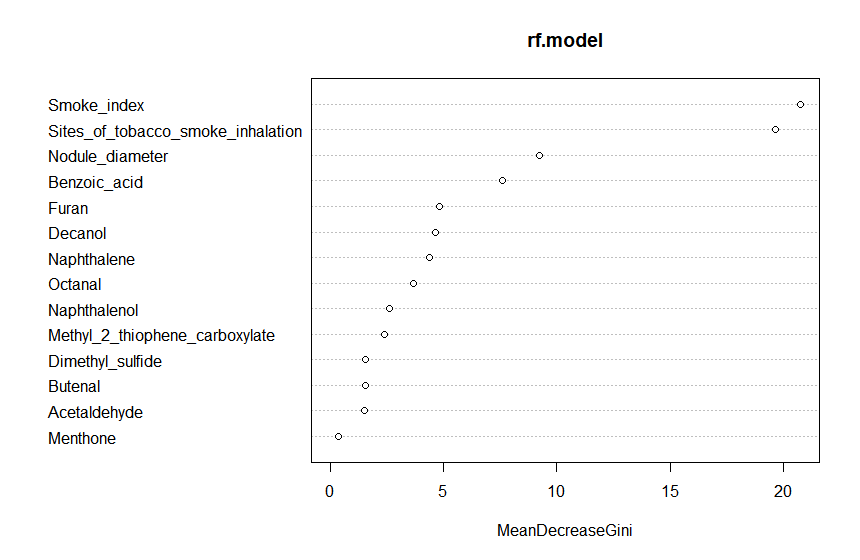


**Supplementary Figure S2. Importance scores of variables incorporated into the breathomics-health examination-lifestyle based predictive model using RF algorithms**. Note: The x-axis indicates the importance of each variable on the outcome. Each point represents a factor.


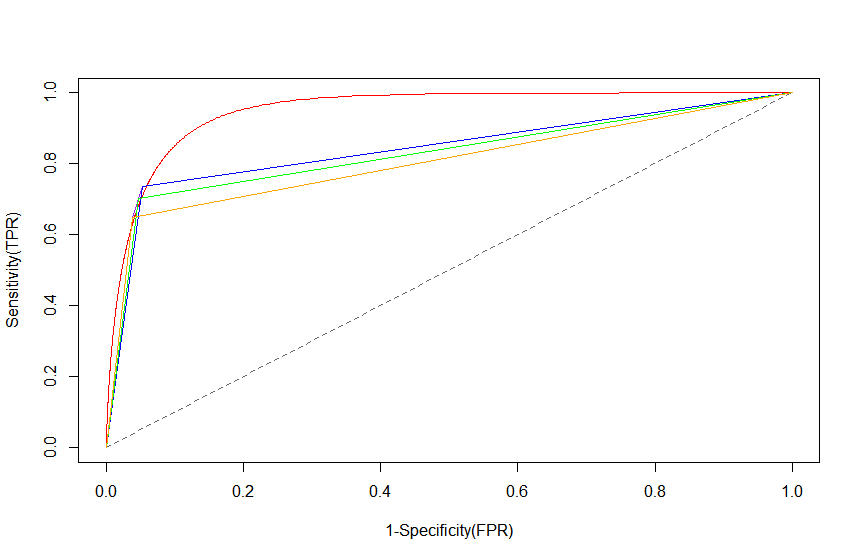


———— LR=0.8482

———— DT=0.8452

———— RF=0.8422

———— KNN=0.8271

———— SVM=0.8055

a)


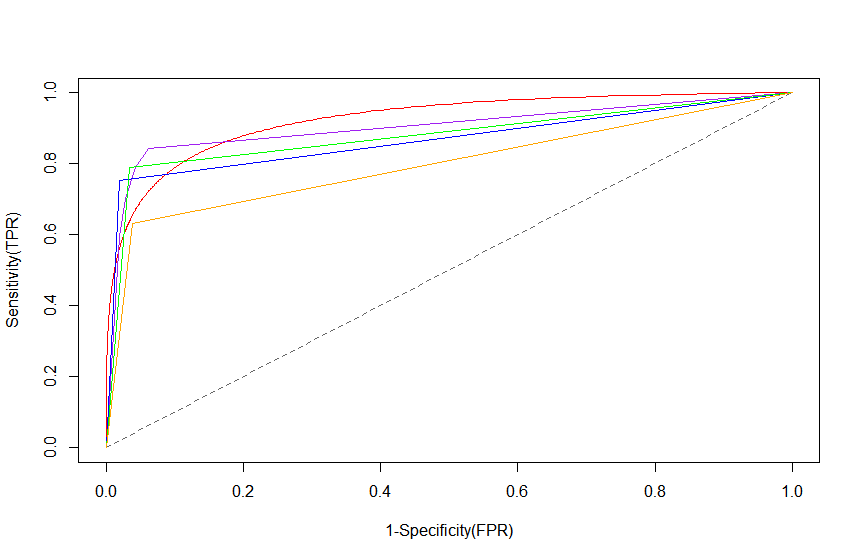

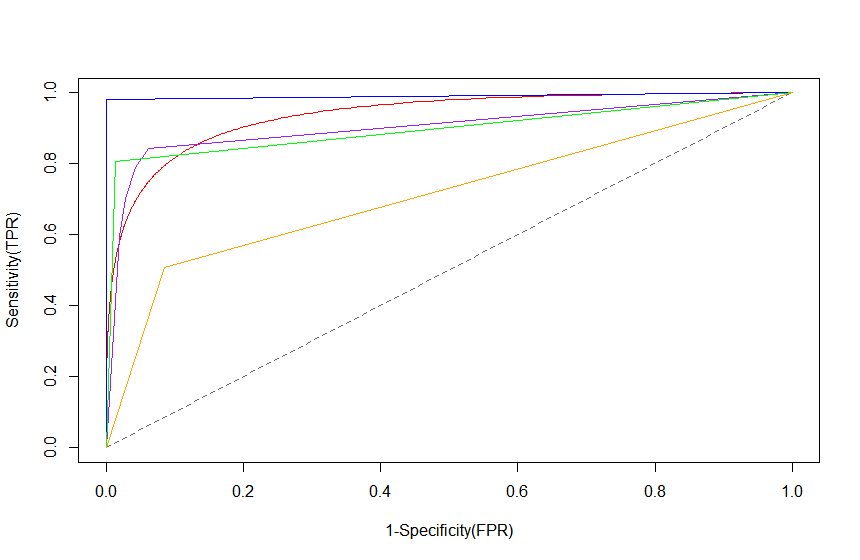


———— RF=0.9912

———— LR=0.9464

———— DT=0.9021

———— KNN=0.8964

———— SVM=0.7115

———— LR=0.9135

———— DT=0.9021

———— KNN=0.8781

———— RF=0.8677

———— SVM=0.7967

c)

b)

**Supplementary Figure S3: AUCs comparison of five machine learning algorithms of a) lifestyle based models, b) health examination-lifestyle based models, c) breathomics-health examination-lifestyle based models.** Abbreviations: AUC: Area under the receiver operating characteristic curve; LR: Logistic regression model; DT: Decision tree model; RF: Random forest model; KNN: K-nearest neighbors model; SVM: Support vector machine model.


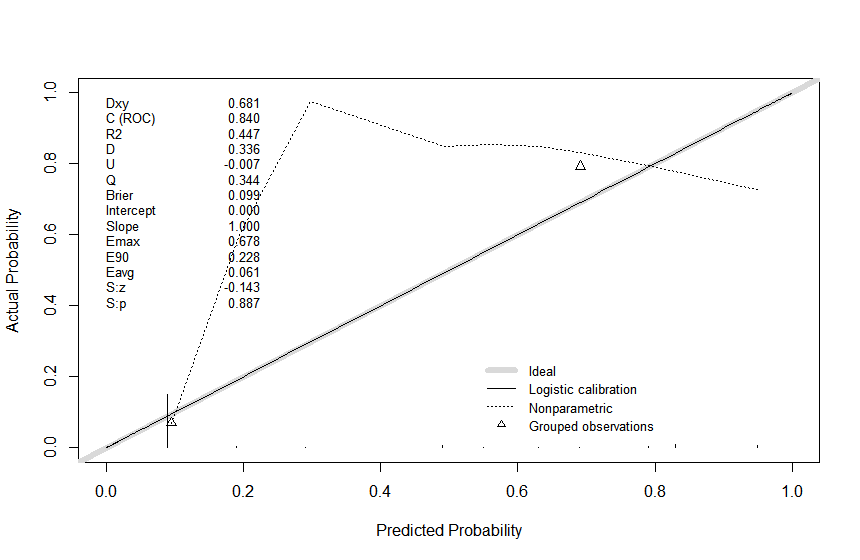


a)


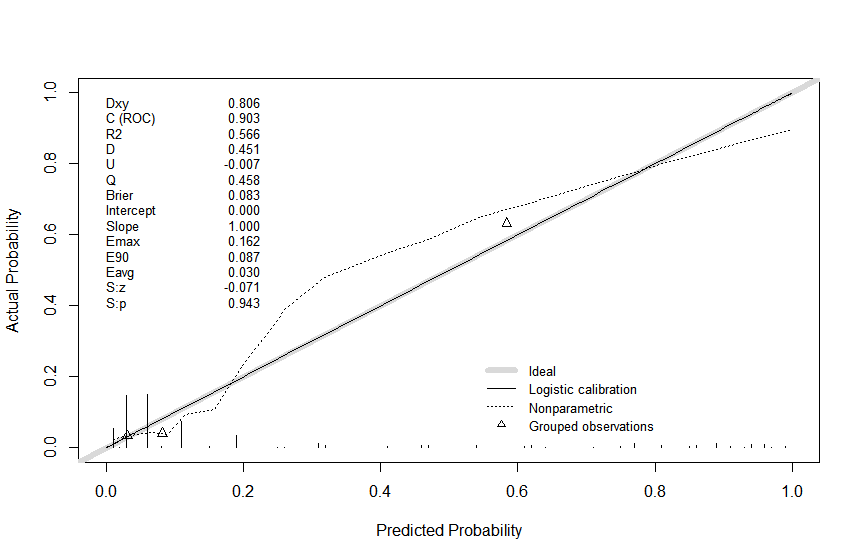


b)


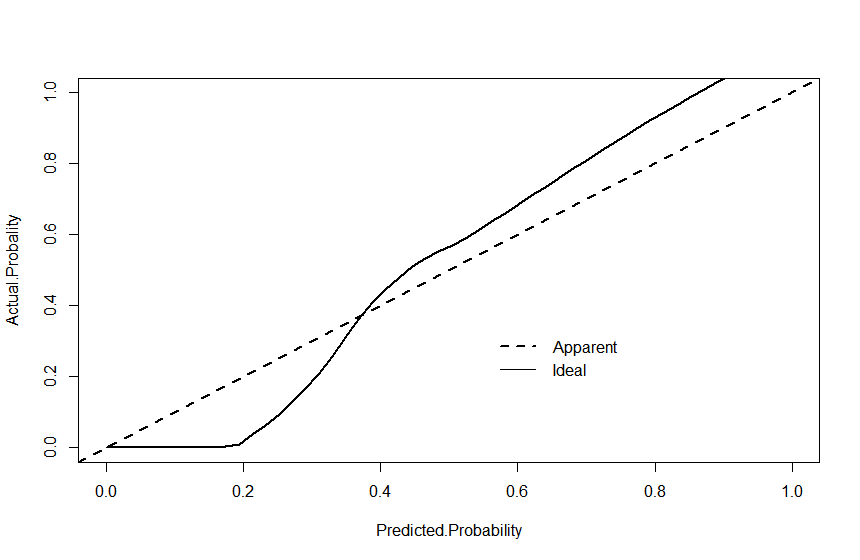


c)

**Supplementary Figure S4: Calibration curve of a) lifestyle based LR model; b) health examination-lifestyle based LR model, and c) breathomics-health examination-lifestyle based RF model.** Note: LR: Logistic regression model; RF: Random forest model.


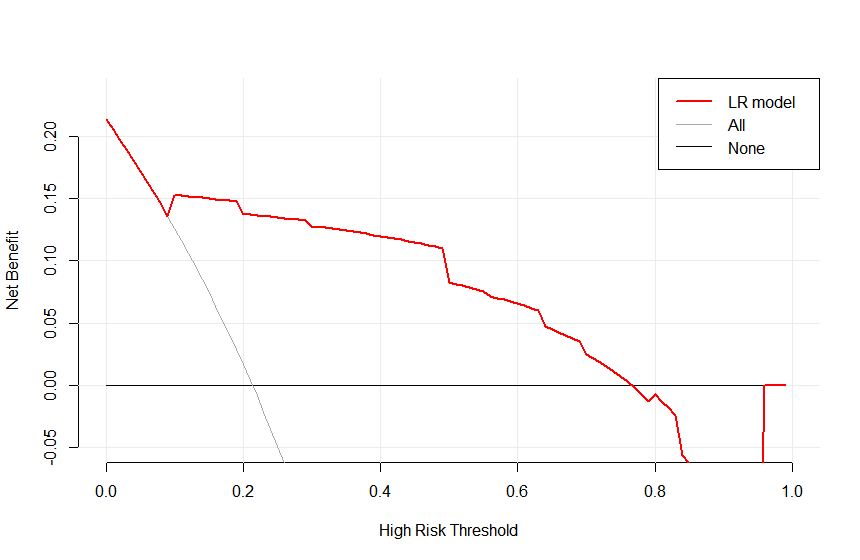


a)


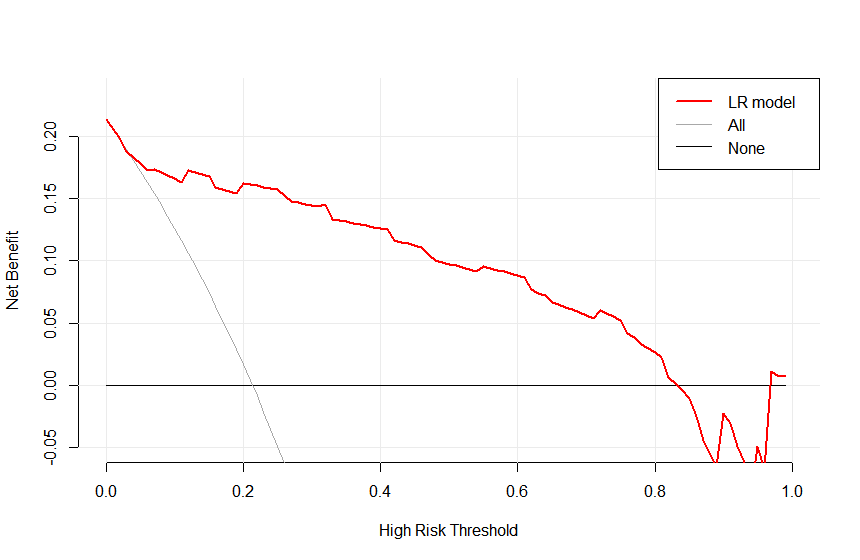


b)


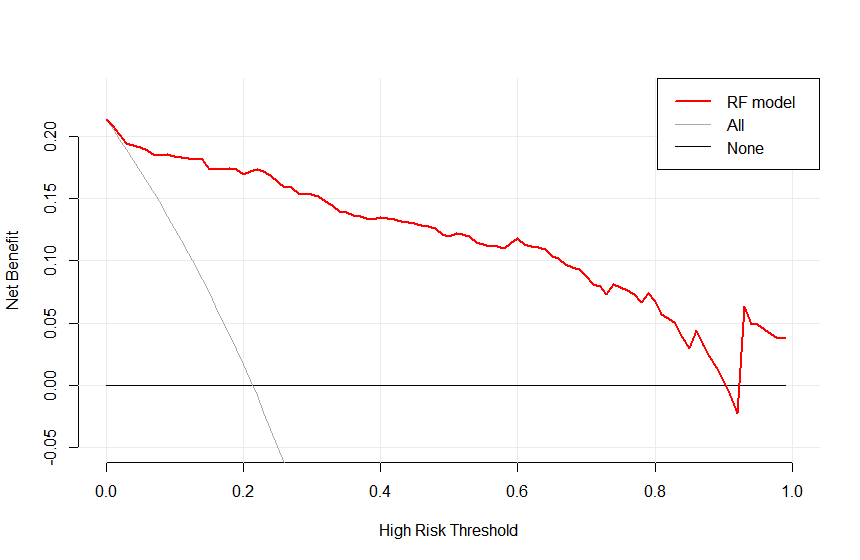


c)

**Supplementary Figure S5: Clinical decision curve of a) lifestyle based LR model; b) health examination-lifestyle based LR model, and c) breathomics-health examination-lifestyle based RF model.** Note: LR: Logistic regression model; RF: Random forest model.
